# Considering single-atom catalysts as photocatalysts from synthesis to application

**DOI:** 10.1016/j.isci.2022.104232

**Published:** 2022-04-08

**Authors:** Haoyue Sun, Rui Tang, Jun Huang

**Affiliations:** 1 School of Chemical and Biomolecular Engineering, Sydney Nano Institute, The University of Sydney, NSW 2006, Australia

**Keywords:** catalysis, materials science, materials chemistry

## Abstract

With the ever-increased greenhouse effect and energy crisis, developing novel photocatalysts to realize high-efficient solar-driven chemicals/fuel production is of great scientific and practical significance. Recently, single-atom photocatalysts (SAPs) are promising catalysts with maximized metal dispersion and tuneable coordination environments. SAPs exhibit boosted photocatalytic performance by enhancing optical response, facilitating charge carrier transfer behaviors or directly manipulating surface reaction processes. In this regard, this article systematically reviews the state-of-the-art progress in the development and application of SAPs, especially the mechanism and performance of SAPs on various reaction processes. Some future challenges and potential research directions over SAPs are outlined at the final stage.

## Graphical abstract

**Figure:**
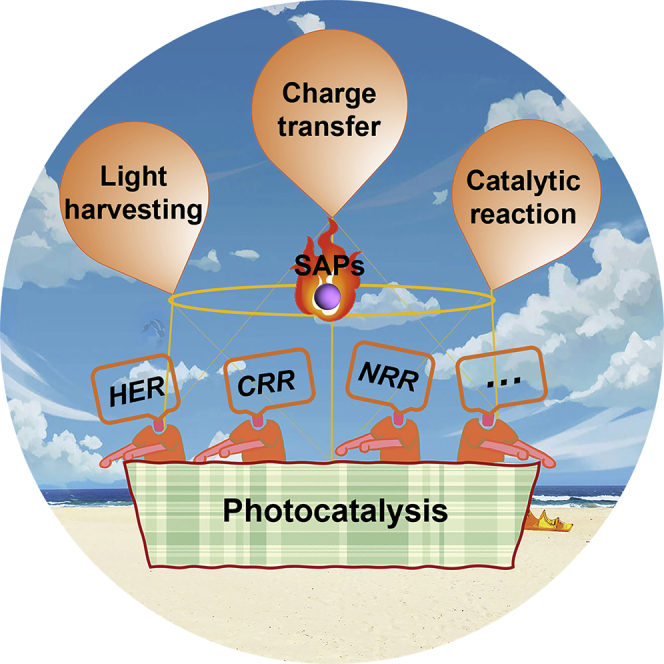

## Introduction

With the increasing demand for energy supply and the ever-worsening climate change, the development of sustainable technologies, such as the solar-driving catalysis process, to generate clean energy and chemicals has been recognized as the most promising way to address this pressing global issue. Photocatalysis has attracted significant attention due to its sustainable characteristics and limited environmental impacts (Lei et al., 2021; Zhu and Zhou, 2019). To date, many semiconductor materials, such as ZnO (Liu et al., 2019b; Shekofteh-Gohari et al., 2018), TiO_2_ (Gao et al., 2020b; Guo et al., 2019), and C_3_N_4_ (Patnaik et al., 2021; Zhu et al., 2021), have been demonstrated with photocatalytic activity. However, as pristine semiconductor materials, their photocatalytic performance is still below the common expectation due to their unsatisfactory photocatalytic processes (i.e., limited light-harvesting capability, severe recombination of photo-induced charge carriers, and poor catalytic selectivity and activity) (Gao et al., 2019a; Zhou et al., 2018). Therefore, it is of great importance to developing novel photocatalysts with an efficient photocatalytic process to perform high-efficient solar-driven chemicals/fuel production.

Recently, single-atom catalysts (SACs), with the active metal species existing as isolated single-atoms (SAs) and stabilized by bonding with the substrate materials or by alloying with another metal (Lai et al., 2019; Zang et al., 2020), have attracted significant attention in the field of photocatalysis due to the unique advantages of SAs, such as maximal metal dispersion and tuneable local coordination environments, exhibiting excellent reaction activity. For the supported metal nanoparticles, it has been widely recognized that their catalytic performance (product selectivity, reaction activity, and the stability of the catalysts, etc.) is strongly influenced by the metal particle size and the local coordination environment between the metal particle and the supporting materials (Campbell et al., 2002; Liu, 2017; Rao et al., 2008; Subramanian et al., 2004). With the particle size decreasing, the surface atoms ratio to the total atoms will sharply increase and result in more unsaturated coordinated surficial metal atoms (Liu, 2017), which could act as the active sites for the reactant molecules' adsorption. When decreased to the small nano-size (2–10 nm) (Halperin, 1986; Li et al., 2021a), the electron energy level of the metal species will split into discrete energy levels (Yang et al., 2015; Yang et al., 2013), which will directly influence the orbital hybridization and charge transfer at the metal/reactant interface (Wang and Lu, 2020).

Generally, SACs possess five main compelling advantages: (1) maximized metal dispersion (Zhang et al., 2021b): as a result, with the same metal loading amount, SACs possess more reactive sites; (2) better product selectivity: all the single-atom sites possess the similar composition and coordination structure, resulting in a uniform catalytic environment (Chen et al., 2021); (3) bridged homogeneous and heterogeneous catalysts (Chen et al., 2018a; Gao et al., 2018): SACs have atomically dispersed metal sites on solid supports and consist of well-defined mononuclear active centers, expected to combine homogeneous and heterogeneous catalysts; (4) unsaturated coordination structure or valence state: the unsaturated coordinated structure or valence state will make the SAs act as the reaction active center and directly participate in the reaction (Chen et al., 2018b); (5) size effect (Hu et al., 2014): the surface free energy of the SAs obviously increases compared with the metal nanoparticles, making them highly active in chemical interactions for reactant molecules (Yang et al., 2013).

**Figure 1:**
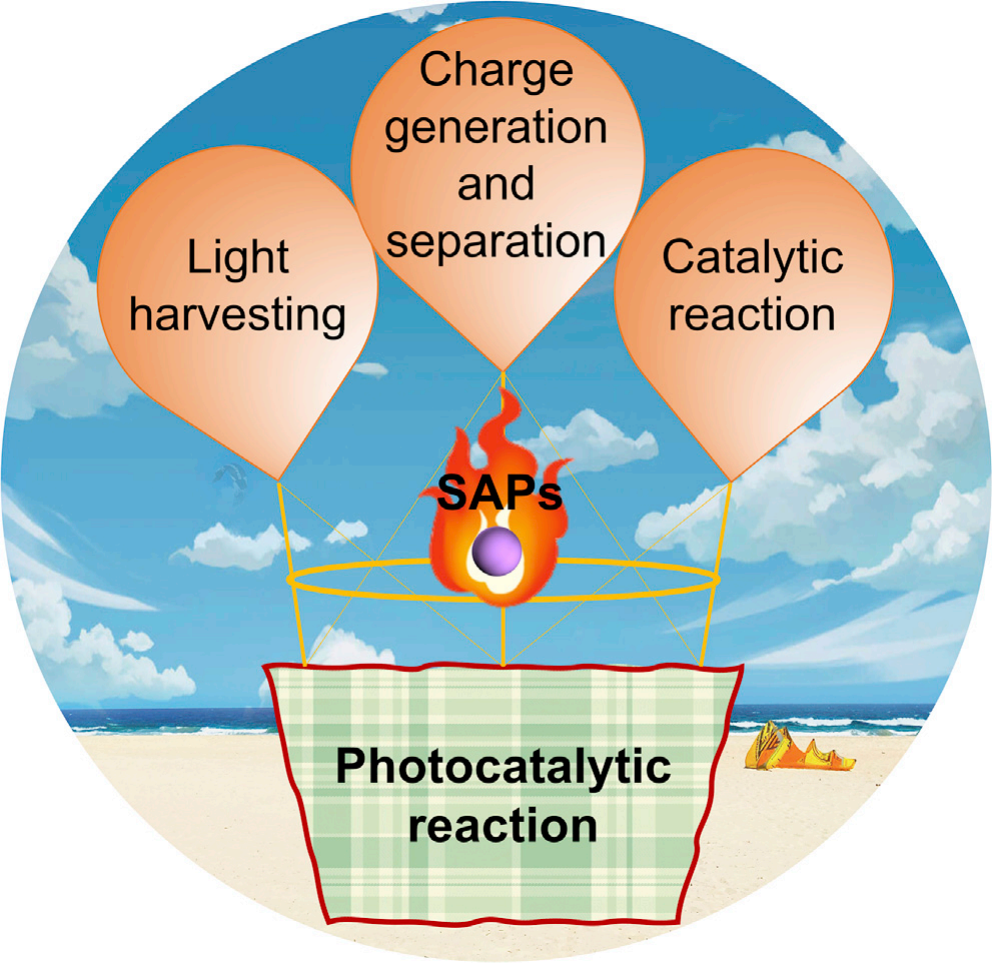
Schematic illustration of SAPs promotes three key characters in photocatalysis

SACs have been at the forefront of catalysis research due to their maximized atom utilization, unique structures, and properties. Among many applications, electrochemical energy conversion is one of the most promising areas, including oxygen evolution (OER), CO_2_ reduction (CRR), etc (Daiyan et al., 2020a). Compared with traditional catalysts, SACs expose more active sites, leading to enhanced electrocatalytic activity. In addition, SACs are also demonstrated with improved selectivity toward the target products. Taking CRR as an example, it was demonstrated that the coordination structure of the SACs was beneficial for reducing the formation energy barrier of the CRR intermediates (e.g., ∗COOH), thereby improving the product selectivity (Daiyan et al., 2020b; Leverett et al., 2021; Leverett et al., 2022). In addition, SACs have also been applied and studied in the photocatalysis fields. It is found that by constructing single-atom photocatalysts (SAPs) with rationally introduced SAs, the overall photocatalysis process could be significantly impacted (Figure 1) (Xia et al., 2021). For instance, it is demonstrated that the introduction of SAs can efficiently alter the electronic structure of the supporting semiconductors, resulting in tuneable optical response behavior (Gao et al., 2020a), and the enhanced charge transfer kinetics of the SAPs can be ascribed to the unique band bending effect (Su et al., 2018). Moreover, due to the tuneable coordination structure between the SAs and the supporting materials, the surface adsorption and activation of the reactant molecules can be boosted (Yang et al., 2020). In this regard, SAPs provide a great platform to regulate the overall photocatalytic reaction process. However, it is also worth to be pointed out that the practical application of SAPs is limited due to the following reasons: (1) the agglomeration of SAs; (2) the structure-performance relationship between the SAPs and the surface photocatalytic reaction is unclear, resulting in vague SAPs-based reaction mechanisms. Therefore, it is of great importance to systematically summarize the current research progress of SAPs for the guidance of future studies on SAPs’ synthesis.

In this review, the unique phenomenon of SAPs brought to the overall photocatalysis processes (i.e., optical response, the separation, and surface reaction process) will be systematically overviewed. As such, the local coordination environment between SAs and the substrates materials will be deeply discussed, and the sequent impact on products' selectivity will be explained. At last, the current challenge and potential research points of SAPs are pointed out. We believe this review will provide guidance knowledge for the future development of novel SAPs catalysts for photocatalytic reaction.

## Principle of photocatalysis

**Figure 2:**
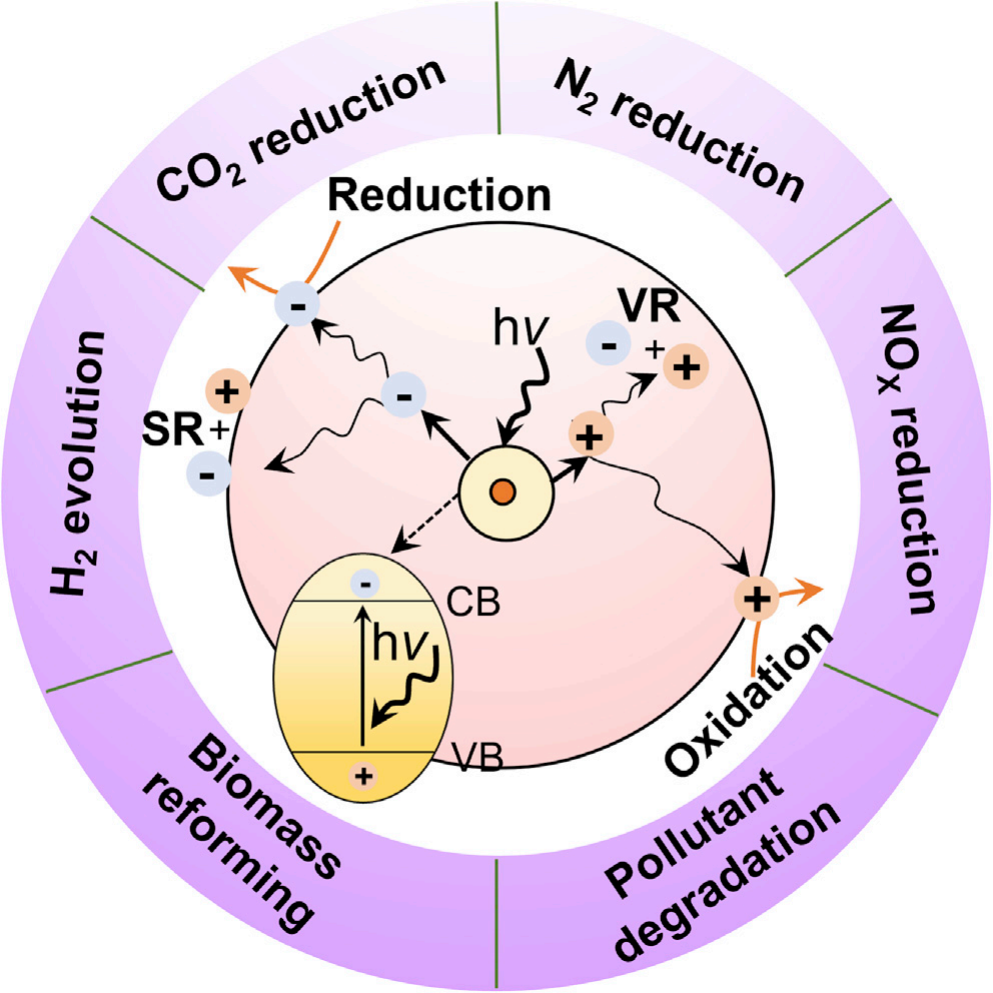
Mechanism of photocatalysis based on traditional semiconductor (SR, surface recombination, and VR, volume recombination)

Photocatalysis is regarded as one sustainable method for solar-driven chemicals/fuels generation. Therefore, within a typical photocatalytic reaction process, it contains three steps to obtain the final redox products, including, (1) the photoexcitation of the photocatalysts and the generation of photo-induced carriers; (2) the spatial separation of photo-induced electrons and holes; (3) the redox of surface-adsorbed reactant molecules. As such, the photocatalytic performance of the catalysts is closely related to the aforementioned three steps: (a) for some wide bandgap semiconductors, such as TiO_2_, ZnO, etc., the narrow optical response performance leads to relatively poor light-harvesting capability and limited amounts of photo-induced carriers; (2) during the spatial separation process, severe carrier recombination processes (both surface recombination and volume recombination) are commonly accompanied, resulting in extra carrier recombination loss; (3) the uncontrollable surface adsorption and activation of the reactant molecule lead to poor product selectivity (Figure 2). Therefore, rationally regulating the aforementioned three steps is of great importance to obtain high-performance photocatalytic processes.

In addition, from the perspective of reaction types, the photocatalysis techniques can be applied to perform various types of solar-driven chemicals/fuels production reactions, including (Wang et al., 2021d), CO_2_ reduction (Tang et al., 2022), N_2_ reduction (Guan et al., 2021), NO_x_ reduction (Zhang et al., 2021d), biomass oxidation reforming (Wang et al., 2021c), and pollutant oxidation degradation (Bui et al., 2021). By virtue of photocatalysis, value-added chemicals and green fuels can be generated in a sustainable and environmental-friendly way. As such, it requires sound development of novel catalysts with favorable adsorption and dissociation modes toward the specific molecules. For instance, in the past decades, noble metal/semiconductor catalysts have been reported as one of the most efficient catalysts for the selective oxidation of aromatics (Chen et al., 2017a). However, the high cost of the catalysts severely limit the scale-up application of such kind of catalysts. In this regard, it makes the development of SAPs of great practical significance. Due to the insufficient understanding of the influence caused by the introduction of SAs on the photocatalysis process, the study on the application of SAPs for the solar-driven chemicals/fuels production is still in its infancy, calling for urgent investigation.

## The synthesis strategies of SAP

When the particle size is reduced to atom level, the metal single atoms with ultra-high surface energy tend to aggregate and form nanoclusters during the synthesis process (Zhao et al., 2020; Zhu et al., 2020). Currently, the reports on SAPs are still limited. Fortunately, the widely reported SACs for other catalysis reactions, such as thermal catalysis, electrocatalysis, etc., can bring us some insights. To date, the synthesis methods of SACs are plentiful, such as impregnation, co-precipitation, chemical vapor deposition (CVD), ion exchange, galvanic replacement, thermochemical method (flame spray pyrolysis [FSP], and pyrolysis of organic materials [metal-organic framework, covalent organic framework, etc.]), atomic layer deposition (ALD), atom trapping, and photochemical reduction. Taking the impregnation method as an example, the prepared substrate material is put into a solution containing SAs precursor. The metal ions are adsorbed on the surface of the substrate material, which are then reduced to produce SACs (Wang et al., 2018). The mass loading of metal SAs for the SACs prepared by the impregnation method is very low, but the procedure is simple (Xia et al., 2022). In addition, the co-precipitation method is also widely used for the preparation of SACs. At least use two cations to form a homogeneous phase in the solution (Qiao et al., 2011). For SACs prepared by co-precipitation, the mass loading must be kept below 1% to prevent agglomeration of the metal particles during calcination or reduction (Xia et al., 2022).

**Figure 3:**
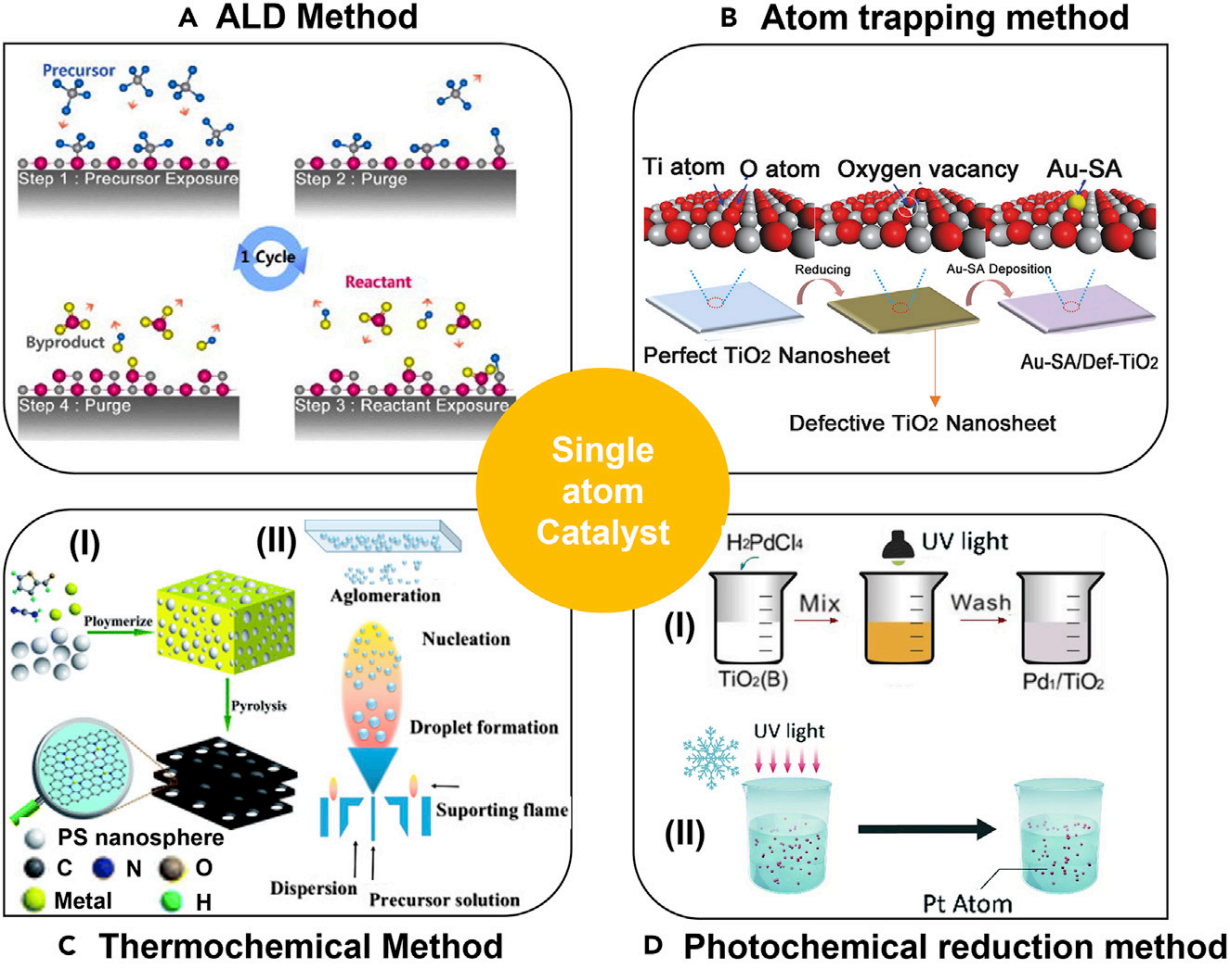
Schematic of four typical SACs synthesis strategies (A) ALD method. Reproduced with permission from Ref (Kim et al., 2009). Copyright 2009, Elsevier. (B) Atom trapping method. Adapted with permission from Ref (Wan et al., 2018). Copyright 2018, Wiley. (C) Thermochemical method ((1) Pyrolysis method. Adapted with permission from Ref (Chen et al., 2020b). Copyright 2020, Royal Society of Chemistry. (2) Flame spray pyrolysis method. Adapted with permission from Ref (Nunes et al., 2019). Copyright 2019, Elsevier.). (D) Photochemical reduction method ((1) Photochemical reduction method. Adapted with permission from Ref (Liu et al., 2016). Copyright 2016, Science. (2) Ice-photochemical reduction method. Adapted with permission from Ref (Wei et al., 2019). Copyright 2019, Royal Society of Chemistry.).

Besides the method mentioned earlier, the following four strategies will be further discussed and compared, including ALD, atom trapping, thermochemical method, and photochemical reduction methods, which are proven to be effective in preventing metal SAs sintering (Figure 3).

### Atomic layer deposition method

**Figure 4:**
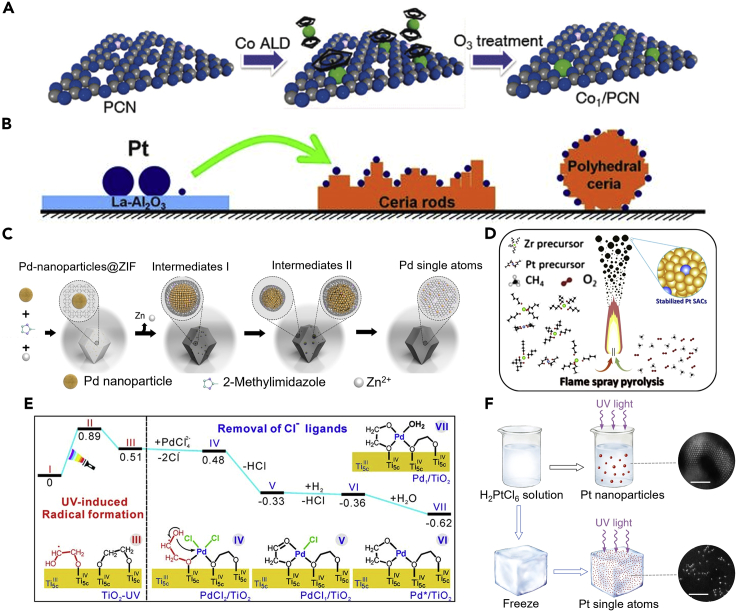
Advanced synthesis strategy of single-atom catalysts (A)The schematic illustration of the synthesis of atom layer decomposition method to synthesis Co single-atom sites. Reproduced with permission from Ref (Cao et al., 2017). Copyright 2017, Wiley. (B) Atom-trapping method to synthesis Pt single-atom sites. Reproduced with permission from Ref (Jones et al., 2016). Copyright 2016, Science. (C) Pyrolysis method to synthesize Pt single-atom sites. Adapted with permission from Ref (Wei et al., 2018). Copyright 2018, Nature. (D) High-temperature flame spray pyrolysis method to synthesize Pt single-atom sites. Adapted with permission from Ref (Ding et al., 2021). Copyright 2021, Elsevier. (E) Energies and models of intermediates and transition states for the photochemical method to synthesize Pd single-atom sites. Reproduced with permission from Ref (Liu et al., 2016). Copyright 2016, Science. (F) Iced-photochemical method to synthesize Pt single-atom sites. Adapted with permission from Ref (Wei et al., 2017). Copyright 2017, Nature.

Atomic layer deposition (ALD) is demonstrated to be a precise method that can deposit the SAs on the surface of the supporting materials by alternately exposing the supports to pulsed vapors of various precursors. As the method is based on chemisorption, deposition occurs only in areas with reactive surface sites (Fonseca and Lu, 2021). Generally, the ALD method includes four steps: (1) exposure to the first precursor; (2) purge of the reaction chamber; (3) exposure to the second reactant precursor; and (4) a further purge of the reaction chamber (Cheng et al., 2016). The morphology, size, density, and loading of the deposited material on the carrier can be precisely controlled by simply tuning the ALD cycle (Cheng et al., 2019). For instance, Cao et al. successfully applied ALD technology and synthesized Co-based catalysts (Co_1_/PCN), which were used in the photocatalytic hydrogen evolution (Figure 4A) (Cao et al., 2017). Specifically, cyclopentadienyl cobalt (Co(Cp)_2_) was used as the cobalt-precursor for the ALD, which was then treated with O_3_ to remove the cyclopentadienyl ligand, resulting in the Co_1_-N_4_ structured SACs. However, nucleation delay and island growth are considered the key issues that need to be optimized (Cheng and Sun, 2017), resulting from the limited functional groups on the surface of supporting materials. Besides, the surface energy difference of metal and support will also bring a challenge to the deposition process. When the surface energy of the support is lower than the free energy of deposited metal, the support cannot be wetted by the deposited metal, which will lead to insufficient adsorption sites and result in an island growth mode finally (Cheng and Sun, 2017).

### Atom trapping method

The atom trapping method is another effective strategy to synthesize the SACs with stably anchored SAs (Wang et al., 2018). Specifically, to plant SAs in the commonly used semiconductors materials, constructing surface defect sites (i.e., C-defect (Chen et al., 2020c; Liu et al., 2021a), O-defect (Cai et al., 2020; Wang et al., 2021e), N-defect (Qin et al., 2021; Zhang et al., 2022a), S-defect (Wang et al., 2020b; Zhang et al., 2021e), metal-defect (Wu et al., 2021; Zhang et al., 2018), etc.) can efficiently capture the SAs. It means that, through defect-engineering strategies, defect sites can be created on the surface of the supporting materials, leading unsaturated coordination environments to the adjacent atoms (Liu et al., 2021a), which can be used as the anchors to trap and stabilize SAs. The necessary synthesis condition of atom trapping requires the mobile metal species and trapping sites on the supports (Qu et al., 2018). Thus, by adjusting the concentration of the surface defects, the SAs loading amount and the catalytic performance of the SACs can be easily regulated (Wang et al., 2019a). For instance, Jones et al. demonstrated that the Pt SAs could be captured and anchored in CeO_2_, forming atomically dispersed Pt_1_/CeO_2_ SACs (Figure 4B) (Jones et al., 2016). In this process, Pt nanoparticles supported on Al_2_O_3_ were aged in the air at 800°C, and the PtO_2_ was released and captured by CeO_2_ in a high-temperature environment, which exhibited quite good thermal stability.

### Thermochemical method

Pyrolysis and flame spray pyrolysis (FPS) are two of the current main thermochemical methods to prepare SACs. The major difference between these two strategies lies in the treatment atmosphere, as the general pyrolysis process calls for inert atmosphere annealing, whereas the FPS process can be processed in air condition (Wang et al., 2012). For pyrolysis, a common method is to adsorb the metal complexes with N-ligand onto the porous support, followed by a pyrolysis step of the metal-organic framework, covalent organic framework (Liu et al., 2019a). For example, it is widely reported that the Co and Fe atoms can coordinate with the N from the support materials to form the Co-N and Fe-N atomic dispersion structure (Wang et al., 2019d; Wu et al., 2019a; Zhang et al., 2021c; Zhu et al., 2017). To synthesize such SACs, generally, the nitrogen/metal precursors and the carbon supports will be pyrolyzed and carbonized under inert atmospheres at a quite high temperature. In addition, Wei et al. also demonstrated that the nanoparticles of noble metals (e.g., Pd, Pt, Au) can be converted to thermal-stable SAs with an annealing temperature of 900°C in the inert atmosphere (Figure 4C) (Wei et al., 2018). With the continuous study on the pyrolysis method, it is confirmed that controlling the temperature change during the pyrolysis process is crucial for the evolution of metal SAs (Wang et al., 2020a).

Unlike pyrolysis, the flame spray pyrolysis method allows the SACs’ synthesis to be carried out in the air conditions, making it a flexible way to produce catalysts with controllable morphology and particle size by adjusting the synthesis conditions (Ding et al., 2021). During the combustion process, the metal salt solution and the solvent of the supporting materials are sprayed into the high-temperature flame simultaneously. The solvent will be vaporized and the metal salt solution will be burned or hydrolyzed in the high-temperature flame, allowing the SAs to be anchored on the supporting materials in one step (Michalow-Mauke et al., 2015; Pongthawornsakun et al., 2015; Thybo et al., 2004). As shown in Figure 4D, Ding et al. reported that, by virtue of the flame spray pyrolysis method, the Pt SAs can be introduced to a series of oxide supports with excellent stability (Ding et al., 2021). Compared with other methods, the flame spray pyrolysis strategy shows unique advantages for the catalysts’ preparation: the flame spray pyrolysis method can synthesize the catalyst quickly and is suitable for scaling up; the SACs produced by the flame spray pyrolysis strategy can exhibit a quite small particle size and well-dispersion (Gavrilović et al., 2018).

### Photochemical reduction method

Photochemical reduction method is a widely used postpreparation strategy to synthesize SACs. This method generally requires that the support materials and the metal salt precursor should be mixed within a reducing agent solution at first. Then the reducing agent will release free radicals under the irradiation of the UV light, reducing and anchoring the SAs to the supporting materials (Li et al., 2018). This method is easy to tailor the SAs loading amount. For instance, by the photochemical strategy, Liu et al. successfully planted the Pd SAs into ultra-thin TiO_2_ nanosheets (Pd_1_/TiO_2_) (Liu et al., 2016), as shown in Figure 4E. The TiO_2_ powder was first dispersed in ethylene glycol (EG) by ultrasonic. Then, the H_2_PdCl_4_ was added as the metal precursor. Under mild UV conditions, the ethylene glycol free radicals can be formed on the surface of the TiO_2_ nanosheets, which promoted the Cl^−^ releasing in the Pd precursor solution and the Pd-O bond formation, resulting in the formation of Pd_1_/TiO_2_. The as-synthesized Pd-TiO_2_ catalysts exhibited excellent catalytic activity for the hydrogenation of C=C bonds and C=O bonds, which was nine times higher than that of commercial Pd catalysts. To further prevent the agglomeration of SAs, Wei et al. demonstrated a novel ice-photochemical reduction method to synthesize Pt SAs (Figure 4F), which were confined and dispersed in the crystal lattice of the supporting materials (Wei et al., 2017). The Pt SAs were obtained by exposing the frozen H_2_PtCl_6_ solution under UV radiation. Then, the molten frozen solution containing Pt atoms was physically mixed with various supports (e.g., TiO_2_) to synthesize Pt-based SACs. Due to the low-temperature feature of the iced-photochemical reduction strategy, it can further avoid the agglomeration of the SAs, which is unavoidable in the room temperature photochemical reduction processes (Lu et al., 2020; Wei et al., 2019). Due to the reduced diffusion rate caused by the low-temperature condition, a hindrance to the agglomeration of SAs thus will be caused, leading to better atomic dispersion of SAs (Lu et al., 2020).

## Current research progress on SAPs

### Possible roles of SAs in the photocatalytic process

Current research on the SAPs have demonstrated that the introduction of SAs will influence the overall three-step photocatalysis process (i.e., photo-excitation, carrier transfer, and surface reaction). Therefore, the related work will be comprehensively overviewed and compared in this section.

#### The optical response range of the photocatalysts

**Figure 5:**
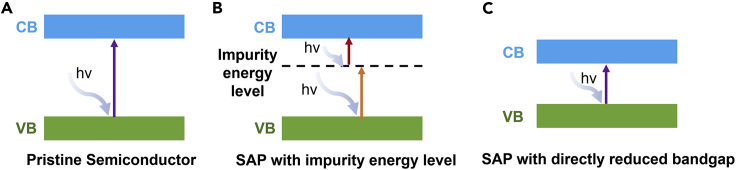
The photo-excitation process of semiconductor photocatalysts (A–C) (A) Pristine semiconductor, (B) SAPs with impurity energy level, and (C) SAPs without directly reduced bandgap.

The optical response capability of the semiconductor catalyst could directly influence the photocatalytic performance, as narrow light-harvesting behaviors will severely limit the incident light utilization efficiency of the catalysts. Previous work demonstrated that the introduction of SAs is effective to enlarge the optical response performance of the pristine catalysts. Some work claimed that the introduction of SAs will cause impurity energy level, thereby broadening the optical response range of the pristine catalysts (Figures 5A–5B) (Yang et al., 2016). In addition, some work also claimed that the introduction of SAs could directly reduce the bandgap of the semiconductor catalysts, rather than introducing impurity energy level, leading to enhanced light absorption capability of the SAPs (Figure 5C) (Yang et al., 2021).

**Figure 6:**
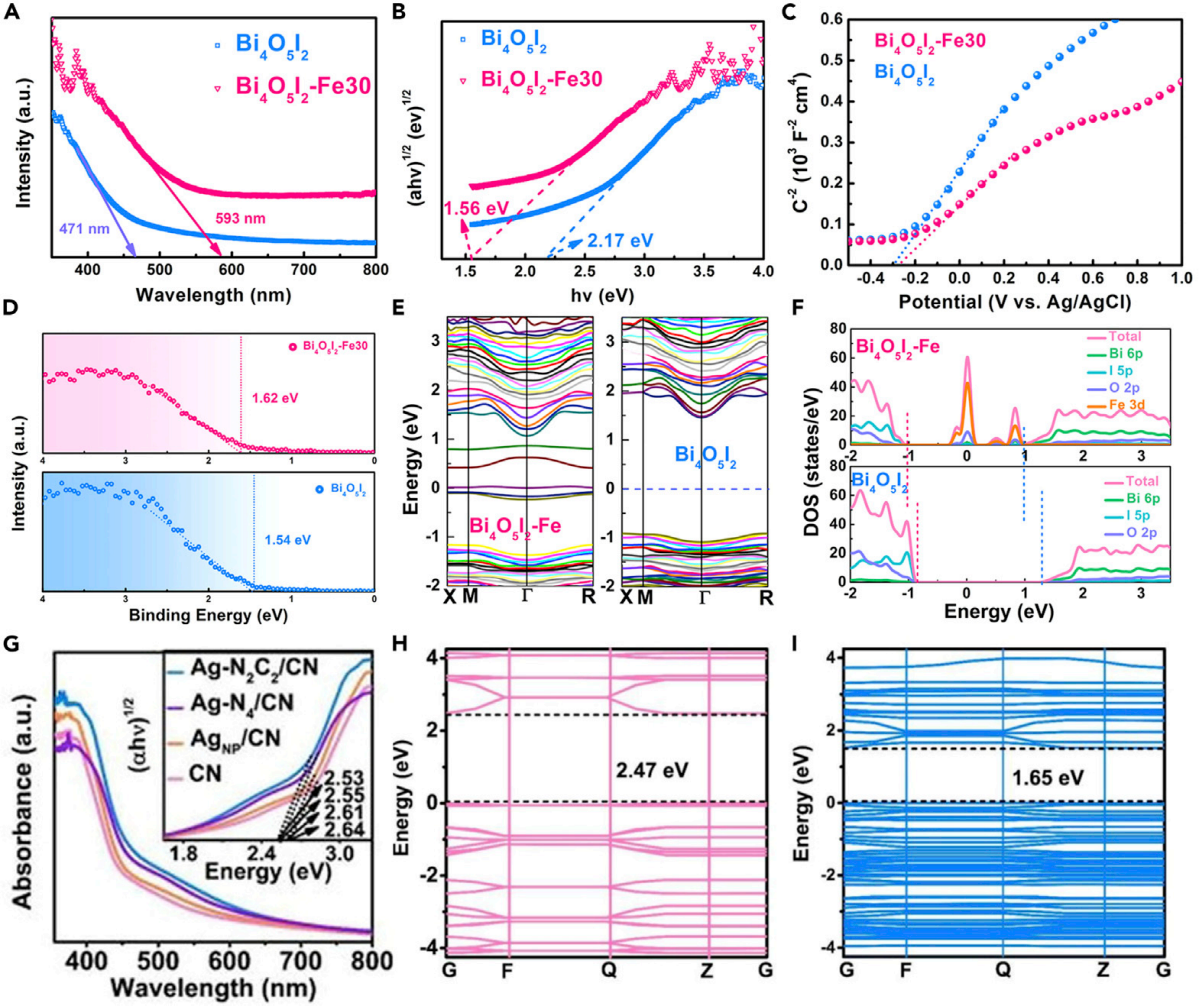
Effect of single-atom modification on the electronic structure of photocatalysts (A–I) Bi_4_O_5_I_2_ and Bi_4_O_5_I_2_-Fe30 of (A) UV-Vis absorption spectra, (B) Tauc plot, (C) Mott-Schottky plots, (D) XPS valence band spectroscopy, (E) DFT calculation for the band structure, and (F) DOS plots. Reproduced with permission from Ref (Jin et al., 2021). Copyright 2021, American Chemical Society. (G) UV-vis absorption spectra and Tauc plots (inset) and DFT calculated the band structures of (H) CN and (I) Ag-N_2_C_2_/CN. Reproduced with permission from Ref (Jiang et al., 2020). Copyright 2020, Wiley.

For instance, Jin et al. proposed SAPs with Fe SAs implanted into the surface of Bi_4_O_5_I_2_ (Bi_4_O_5_I_2_-Fe30) (Jin et al., 2021). As such, Fe SAs were considered as the dopant to replace the Biatoms in Bi_4_O_5_I_2_, which generated impurity energy levels within the bandgap of Bi_4_O_5_I_2_, leading to a broadened light-harvesting range toward the Bi_4_O_5_I_2_-Fe30 SAPs. From the ultraviolet-visible (UV-Vis) diffuse reflectance spectra, a red-shifting light absorption trend was demonstrated when the Fe SAs were introduced to the Bi_4_O_5_I_2_ (Figure 6A). The Tauc plot diagram in Figure 6B also confirmed that the bandgap values reduced from 2.17 eV of Bi_4_O_5_I_2_ to 1.56 eV of Bi_4_O_5_I_2_-Fe30. The aforementioned evidence indicated that the light absorption and electronic structure of pristine semiconductors can be modulated by incorporating SAs. By virtue of both experimental (Figures 6C and 6D) and theoretical calculation (Figures 6E and 6F), the energy band structure of Bi_4_O_5_I_2_ and Bi_4_O_5_I_2_-Fe30 were further determined. It was confirmed that, after the introduction of Fe SAs, impurity energy levels appeared near the conduction band of Bi_4_O_5_I_2_, therefore enlarging the optical response range of Bi_4_O_5_I_2_. Li et al. also demonstrated Ru SAs doped monolayered TiO_2_ nanosheets (Ru_1_/TiNS) and confirmed that after Ru_1_ SAs inducing, an isolated impurity energy level was formed, broadening the optical absorption range up of the Ru_1_/TiNS to 700 nm (Li et al., 2020b).

Moreover, it was reported that the introduction of SAs could directly reduce the bandgap of the catalysts, leading to an enlarged light absorption range. For instance, Jiang et al. developed a novel Ag SAs incorporated carbon nitride photocatalyst (Ag-N_2_C_2_/CN) with the Ag-N_2_C_2_ configuration (Jiang et al., 2020). As shown in Figure 6G, with the Ag-N_2_C_2_ and Ag-N_4_ coordination structure, the optical response range of the obtained catalysts can be efficiently expanded. The inset in Figure 6G showed a reduced narrowed bandgap after incorporating Ag SAs. The DFT calculation also confirmed the introduction of Ag SAs would lead to the directly reduced bandgap but no impurity energy level (Figures 6H and 6I).

#### Promoting the charge separation and transfer of the photocatalysts

The efficient spatial separation and transfer of photogenerated carriers are important for performing high-efficient photocatalytic reactions. But for pristine semiconductor catalysts, severe carrier recombination is unavoidable (Liu et al., 2020). This unavoidable energy loss before the surface reaction would result in relatively low photocatalytic activity. The introduction of SAs is also demonstrated to contribute to efficient carrier separation (Dong et al., 2021b; Xiao et al., 2020). According to the previous studies, generally, when the metal nanoparticle is in contact with the semiconductor, a Schottky barrier is generated on behalf of the energy level matching of the metal and the semiconductor. After the electronic equilibrium is established at the interface, the photogenerated electrons in the semiconductor will pass through the Schottky barrier and transfer to the metal particles (Gao et al., 2020a). This charge transfer behavior across the metal-semiconductor interface can be inherited by the SAPs (Gao et al., 2020a; Gopalakrishnan et al., 2021). It was demonstrated that, when the metal SAs were contacted with the semiconductor substrate, efficient charge transfer behaviors could be expected due to the interfacial barrier (Meng et al., 2019; Wang et al., 2019a). In addition, it was considered that, by introducing SAs, the charger transfer distance between the light-harvesting units and the photocatalytic sites could be shortened (Gao et al., 2020a).

**Figure 7:**
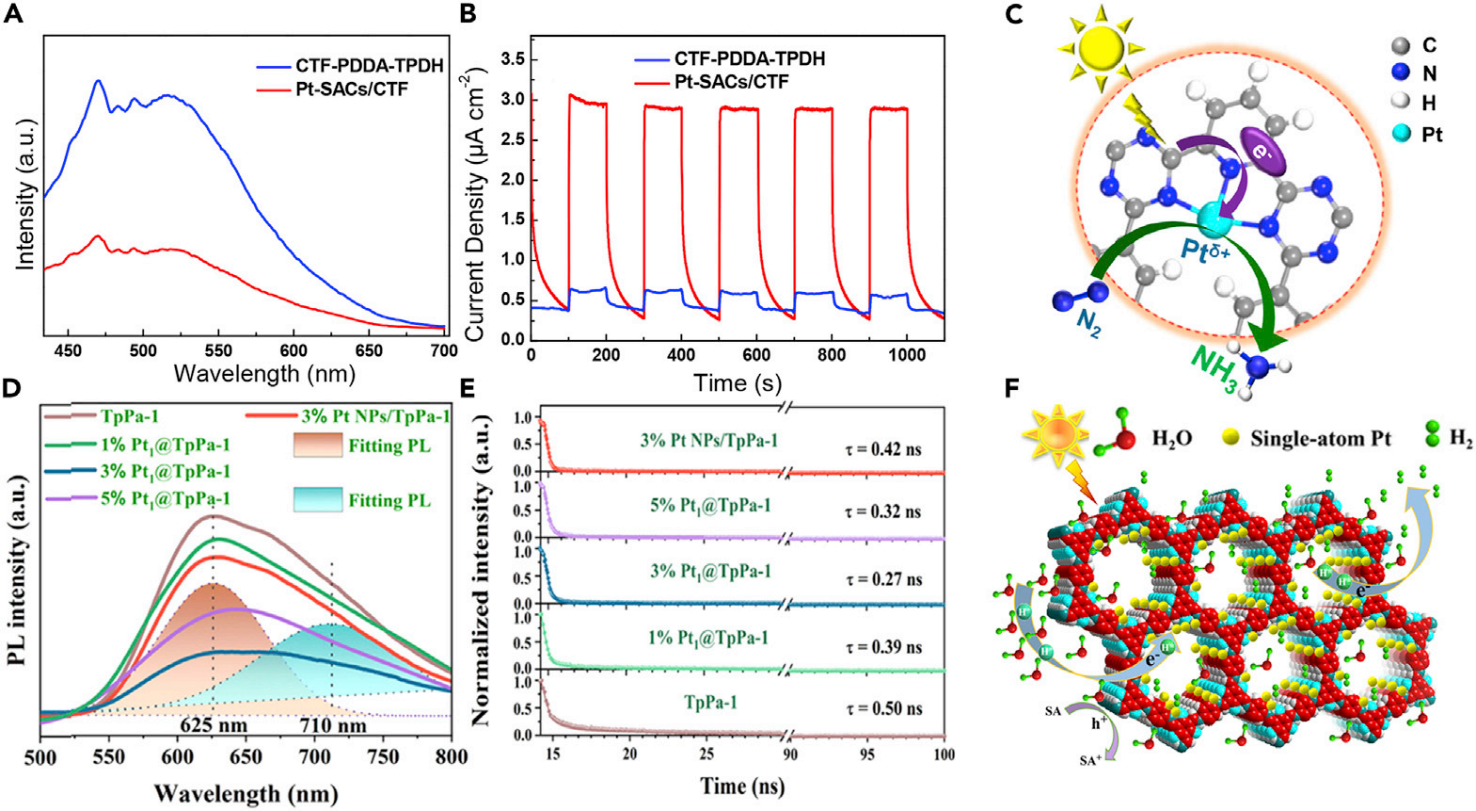
Effect of single-atom modification on the charge separation and transfer of photocatalysts (A–C) (A) PL spectra, (B) chronoamperometry curves of CTF-PDDA-TPDH and Pt-SACs/CTF catalysts, and (C) photocatalytic N_2_ fixation mechanism of the Pt-SACs/CTF catalyst, respectively. Reproduced with permission from Ref (Li et al., 2020a). Copyright 2020, American Chemical Society. (D–F) (D) PL spectra excited by a wavelength of 365 nm, (E) TRPL spectra, (F) schematic illustration of photocatalytic H_2_ over Pt_1_@TpPa-1. Reproduced with permission from Ref (Dong et al., 2021a). Copyright 2021, American Chemical Society.

For instance, Li et al. demonstrated that, by incorporating Pt SAs into ultra-thin covalent triazine framework nanosheets (Pt-SACs/CTF) with unique Pt-N_3_ structure, the carrier migration in the Pt-SACs/CTF catalysts can be efficiently enhanced (Li et al., 2020a). It was demonstrated that the electrons captured by the Pt SAs were subsequently utilized for the nitrogen fixation reaction. In sharp contrast with the ultra-thin covalent triazine framework nanosheets (CTF-PDDA-TPDH), the Pt-SACs/CTF catalysts showed a weaker PL intensity (Figure 7A), indicating the suppressed carrier recombination in Pt-SACs/CTF. The photoresponse of the Pt-SACs/CTF and CTF-PDDA-TPDH catalysts were further studied by the chronoamperometry curves under chopped optical illumination (Figure 7B). The CTF-PDDA-TPDH nanosheet catalysts showed lower photocurrent density, whereas the photocurrent density increased obviously after incorporating Pt SAs. These results elucidated the enhanced carrier separation efficiency of the Pt-SACs/CTF catalyst. The proposed carrier transfer mode was shown in Figure 7C. Under visible light irradiation, the CTF-PDDA-TPDH nanosheets were excited with the electrons on the CB of the CTF-PDDA-TPDH nanosheets transferred to the Pt SAs, bringing about the enhanced carrier separation. The electrons captured by the Pt SAs were then consumed to reduce the N_2_ molecule into NH_3_.

Similarly, Dong et al. demonstrated that the Pt SAs could be anchored on the covalent organic framework (COF) catalysts, linked by β-ketoenamine (Pt_1_@TpPa-1) (Dong et al., 2021a). As-synthesized photocatalysts showed high activity (99.86 mmol g_pt_^−1^ h^−1^) and selectivity (100%) for H_2_ evolution, which were attributed to the successful anchoring of Pt SAs to facilitate the transfer efficiency of photogenerated electrons. The photoluminescence (PL) spectra exhibited two fitting peaks centered at 625 and 710 nm in Figure 7D. And the PL peak at 625 and 710 nm was attributed to the bandgap radiative recombination and the π-π interaction between the COF and β-ketoenamine layers, respectively. Moreover, these two emissions peaks of Pt_1_@TpPa-1 were quenched significantly compared with TpPa-1, due to the interfacial charge transfer from TpPa-1 to Pt SAs. The charge transfer behavior was further probed through the time-resolved PL (TRPL) decay spectra (Figure 7E). It was shown that the anchoring of Pt SAs (3% Pt_1_@TpPa-1) led to a shorter lifetime (0.27 ns) compared with TpPa-1 (0.50 ns), which was attributed to the addition of Pt SAs in the COF. The possible charge transfer behavior and reaction routes are illustrated in Figure 7F. As a result, the Pt_1_@TpPa-1 provided more photocarriers for the subsequent surface photocatalysis reactions, thus improving the photocatalytic performance. The protons (H^+^) produced by the dissociation of H_2_O were then reduced to the transitional state (H∗) and finally evolved into H_2_. The aforementioned result implied that the Pt SAs could facilitate the efficient migration of photoelectrons, thus improving the efficiency of the photocatalytic reactions.

#### Enhancing the surface reaction process

**Figure 8:**
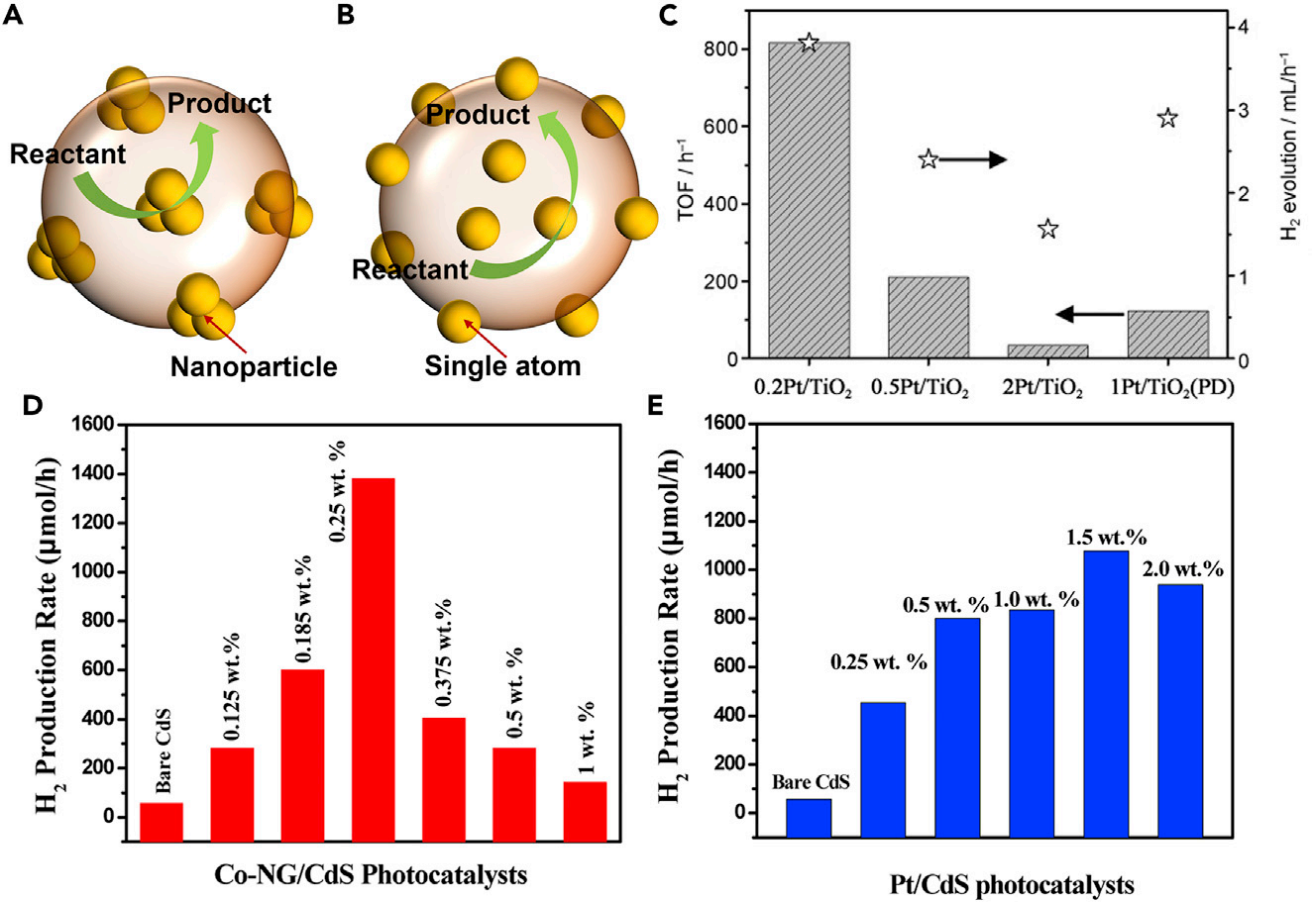
Surface active sites of photocatalysts (A and B) (A) Metal nanoparticles and (B) metal single atoms loaded on the support. (C) TOF and H_2_ evolution of 0.2Pd/TiO_2_, 0.5Pd/TiO_2_, 2Pd/TiO_2_, and 1Pd/TiO_2_ (PD). Reproduced with permission from Ref (Xing et al., 2014). Copyright 2014, Wiley. (D and E) (D) Hydrogen production rate over Co-NG/CdS photocatalysts with different Co-NG loadings and (E) hydrogen production rate of different Pt loadings on CdS (Pt/CdS). Reproduced with permission from Ref (Zhao et al., 2017). Copyright 2017, American Chemical Society.

Besides affecting the photocatalysts' light-harvesting and charge transfer capability, it is demonstrated that the introduced SAs could also act as the reaction active sites and directly participate in the reaction (Liu et al., 2021a; Wang et al., 2021b). Compared with general metal nanoparticle/semiconductor catalysts, the SAPs, maximizing the atomic utilization, exhibit boosted reaction active sites and consequent excellent photocatalytic performance (Figures 8A and 8B) (Jiao et al., 2021; Wang et al., 2019e; Zhang et al., 2022a). It is generally considered that the surface reaction process is strongly influenced by the geometric effect and the electronic structure of the catalysts (Gao et al., 2020a). Constructing SAPs provides an effective method to manipulate the local coordination environments of the SAs, offering an easy way to regulate the adsorption/activation mode of the reactant as the surface of the catalysts. In addition, as the SAs offered a large number of unsaturated coordination centers, numerous reaction active sites could be provided for the surface reaction process (Wang et al., 2019b).

##### Enhancing the reaction active sites

Currently, metal oxides (TiO_2_, ZnO, etc.) and metal sulfides (CdS, MoS_2_, *etc.*) are widely studied as the photocatalysts (Kumar et al., 2021; Kusiak-Nejman et al., 2021; Premaratne et al., 2004). As discussed in section atomic layer deposition method, by creating defect sites (i.e., O-defect, S-defect), the metal-oxides- and metal-sulfides-based SAPs can be easily synthesized by the atom trapping methods. Due to the synergistic effect of the SAs and the adjacent defect sites, it is reported that enormous reaction active sites could be provided.

For instance, Xing et al. successfully loaded various SAs (i.e., Pt, Pd, Ru, Rh, etc.) onto the TiO_2_ substrate for photocatalytic hydrogen evolution reactions (HER) (Xing et al., 2014). The turnover frequencies (TOFs) of 0.2Pt/TiO_2_ photocatalytic hydrogen production was about 24 and 6 times higher than that of 2Pt/TiO_2_ and 1Pt/TiO_2_ (PD, denoted nanoparticles), respectively (Figure 8C). It was found that the same phenomenon also existed in some other SAPs (Pd, Ru, and Rh), confirming the introduction of SAs could provide extra reaction active sites compared with bulk catalysts. In addition, Wu et al. successfully introduced various concentrations of Pt SAs onto the TiO_2_ nanotubes in different concentrations of HPtCl_4_ solution (2–0.0005 mM) and applied them for the photocatalytic HER (Wu et al., 2021). It was found that compared with Pt nanoparticles, the Pt SAPs also exhibited better HER performance.

For metal-sulfides-based SAPs, similar results were evidenced. For instance, Zhao et al. reported Co SAs/N-doped graphene-modified CdS (Co-NG/CdS) (Zhao et al., 2017), exhibiting efficient photocatalytic HER performance. The 0.25 wt% of Co-NG/CdS showed an H_2_ evolution rate of 1077 μmol h^−1^, which was 1.3 times higher than that of the Pt-NPs/CdS photocatalyst (1382 μmol h^−1^), confirming the contribution of SAs on the reaction active sites (Figures 8D–8E). Moreover, the turnover numbers (TONs) were calculated to be 58.2 and 474.764 for the CdS and Co-NG/CdS, respectively. For the 0.25 wt% Co-NG/CdS photocatalyst, the TOF was approximately 8.8 s^−1^. These TON and TOF values show that, under the same reaction conditions, the SAPs showed better reaction activity than the metal nanoparticle/semiconductor catalysts.

##### Contributing to better product selectivity

As the coordination environment of the active atoms in SAPs can be flexibly regulated, it affords us an effective effort to regulate the reactant adsorption/activation modes on the catalysts’ surface, thereby altering the reaction pathway and finally achieving high product selectivity in the photocatalytic process (Gao et al., 2020a; Wang et al., 2021g). As discussed in section principle of photocatalysis, photocatalysis techniques have been widely applied to various solar-driven chemicals/fuels generation, including HER (Alarawi et al., 2019; Zhang and Guan, 2020), CRR (Gao et al., 2018; Wang et al., 2019c), nitrogen reduction reaction (NRR) (Huang et al., 2018; Li et al., 2020a), etc. Table 1 overviewed some recent research on SAPs for the inorganic photocatalytic reactions.

**Table 1:** Comparison list of recently reported SAPs for inorganic photocatalytic reactions

Reaction	SAP	SA	Light intensity	Yield rate	Ref.
HER	Ag_1_/CN	Ag	300 W xenon lamp (*λ* > 400 nm)	1688.9 mmol h^−1^ g^−1^_metal_	(Yan et al., 2022)
	CuSA-TiO_2_	Cu	Xe lamp (325 W/m^2^)	101.7 mmol g^−1^ h^−1^	(Zhang et al., 2022b)
CRR	RuSA-mC_3_N_4_	Ru	34 W Blue LED (700 nm > λ > 420 nm)	250 μmol g^−1^ h^−1^ (CH_3_OH)	(Sharma et al., 2021)
	Co-MOLs	Co	300 W Xe lamp (λ > 420 nm)	464.1 μmol g^−1^ h^−1^ (CO)	(Zhang et al., 2021a)
NRR	La/MoO_3-x_	La	300 W Xe lamp 1.73 W/cm^2^ (λ > 420 nm)	209.0 μmol h^−1^ g ^−1^	(Liu et al., 2022)
	Ru-SA/H_*x*_MoO_3-*y*_	Ru	300 W Xe lamp (λ > 420 nm)	4.0 mmol h^−1^ g^−1^	(Yin et al., 2021)

**Figure 9:**
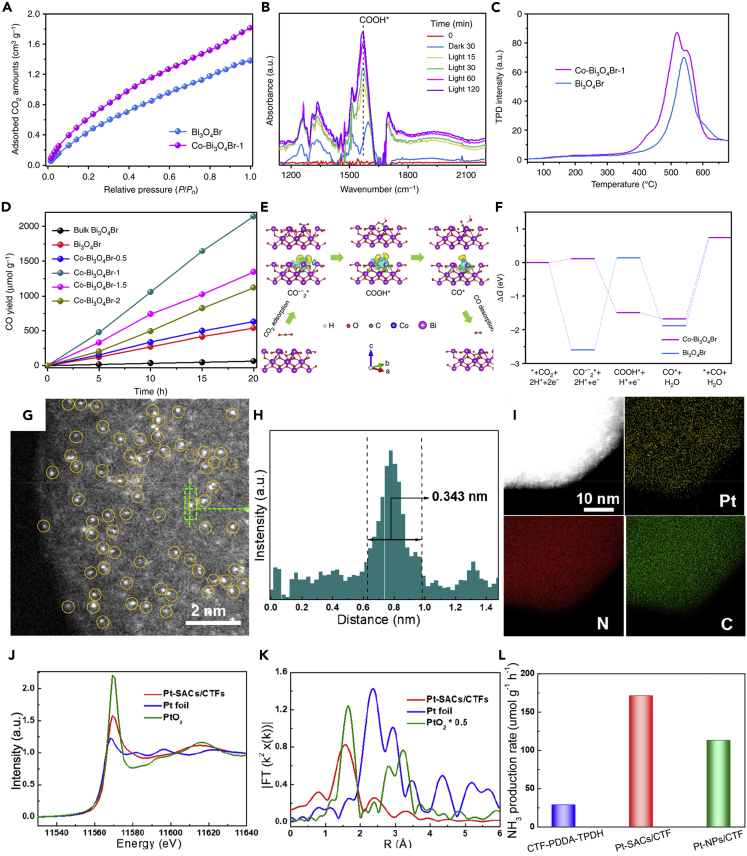
Single-atom photocatalysts for inorganic photocatalytic reactions (A–F) (A) CO_2_ adsorption isotherms of Bi_3_O_4_Br and Co-Bi_3_O_4_Br-1, (B) *in situ* FTIR spectra for the CO_2_ reduction process on the Co-Bi_3_O_4_Br-1, and (C) CO TPD spectra of Bi_3_O_4_Br and Co-Bi_3_O_4_Br-1, (D) Photoreduction of CO_2_ into CO over Bi_3_O_4_Br and Co-Bi_3_O_4_Br, (E) schematic representation of photoreduction CO_2_ mechanism on the Co-Bi_3_O_4_Br, and (F) free-energy diagrams of photocatalytic reduction of CO_2_ to CO for the Bi_3_O_4_Br and Co-Bi_3_O_4_Br. Reproduced with permission from Ref (Di et al., 2019). Copyright 2019, Nature. (G–L) (G) HAADF-STEM image of Pt-SACs/CTF catalyst, (H) corresponding intensity profiles for Pt SAs, (I) EDS mapping images of the Pt-SACs/CTF catalyst, (J) XANES spectra for Pt L3-edge, (K) EXAFS spectra for Pt L3-edge, and (L) NH_3_ production rate of CTF, Pt-SACs/CTF, and Pt-NPs/CTF catalyst. Reproduced with permission from Ref (Li et al., 2020a). Copyright 2020, American Chemical Society.

For photocatalytic CO_2_ reductions, it was demonstrated that the presence of some SAs can enhance the adsorption of CO_2_ molecules, stabilize the photocatalytic CO_2_ reduction intermediates, and accelerate the CO desorption, thereby achieving better CO selectivity in the photocatalytic process (Zhang et al., 2020). For instance, Di et al. demonstrated that, by replacing Bi^3+^ with Co SAs, the CoBi_3_O_4_Br atomic shell could be negatively charged, which facilitated the adsorption of CO_2_, as shown in Figure 9A (Di et al., 2019). By virtue of *in-situ* Fourier transform infrared spectroscopy (FTIR), it allowed insight into the reaction intermediates of photocatalytic CO_2_RR (Figure 9B). The, peaks at 1256, 1337, and 1508 cm^−1^ were attributed to the CO_2_^−^, symmetrical O-C-O extended bidentate carbonate (b-CO_3_^2−^) and monodentate carbonate (m-CO_3_^2−^) groups, respectively. The increasing peak intensity at 1567 cm^−1^ was attributed to the COOH∗ intermediate, which was an important intermediate for the formation of CO. Finally, the CO desorption was also considered to be an important factor in determining the comprehensive photocatalytic efficiency. The temperature-programmed desorption (CO-TPD) curves in Figure 9C demonstrated that Co-Bi_3_O_4_Br-1 possessed a lower initial CO desorption temperature, indicating that as-formed CO∗ could be easily removed from Co-Bi_3_O_4_Br-1 surface and higher CO yield rates (Figure 9D). Therefore, the introduction of Co SAs could promote the adsorption of CO_2_ molecules and reduce the activation energy barrier of CO_2_ by stabilizing the COOH∗ intermediate and adjusting the rate-limiting step to CO∗ desorption (Figures 9E–9F), thus exhibiting excellent photocatalytic activity and selectivity.

The SAPs have also been applied for photocatalytic NRR. For instance, Li et al. successfully anchored Pt SAs to the ultra-thin CTF nanosheet (Pt-SAC/CTF) (Li et al., 2020a). The high-angle annular dark-field scanning transmission electron microscopy (HADDF-STEM) images of the obtained catalysts were shown to confirm the appearance and atomic diameter distribution of the Pt-SAC/CTF catalyst (Figures 9G and 9H). The even dispersion of Pt, C, and N atoms corresponding to the EDS mapping image could be observed in Figure 9I. The electronic state of the Pt species in the Pt-SAC/CTF catalyst was explored by the X-ray absorption near-edge structure analysis (XANES) (Figure 9J). The white line intensity of the Pt-SAC/CTF was lower than that of PtO_2_, but higher than that of Pt foil, indicating that the unoccupied density lay between PtO_2_ and Pt foil. The extended X-ray absorption fine structure (EXAFS) spectrum confirmed the same coordination structure of the Pt-N_3_ site in the ultra-thin CTF-PDDA-TPDH nanosheets. The Fourier transform EXAFS (FT-EXAFS) shown in Figure 9K showed a main peak at 2.34 Å, which corresponded to the metal Pt bond of the standard Pt foil. The sharp peak at 1.57 Å for the Pt-SAC/CTF catalyst indicated that the Pt presented as SAs in the Pt-SAC/CTF catalyst. Then under the visible light radiation, the photocatalytic N_2_ immobilization experiment was carried out. The average NH_3_ production rate of the Pt-SAC/CTF catalyst was 171.40 μmol g^−1^ h^−1^ (Figure 9L), which was 6 times and 1.5 times higher than that of the CTF-PDDA-TPDH and Pt-NPs/CTF catalysts, respectively.

**Figure 10:**
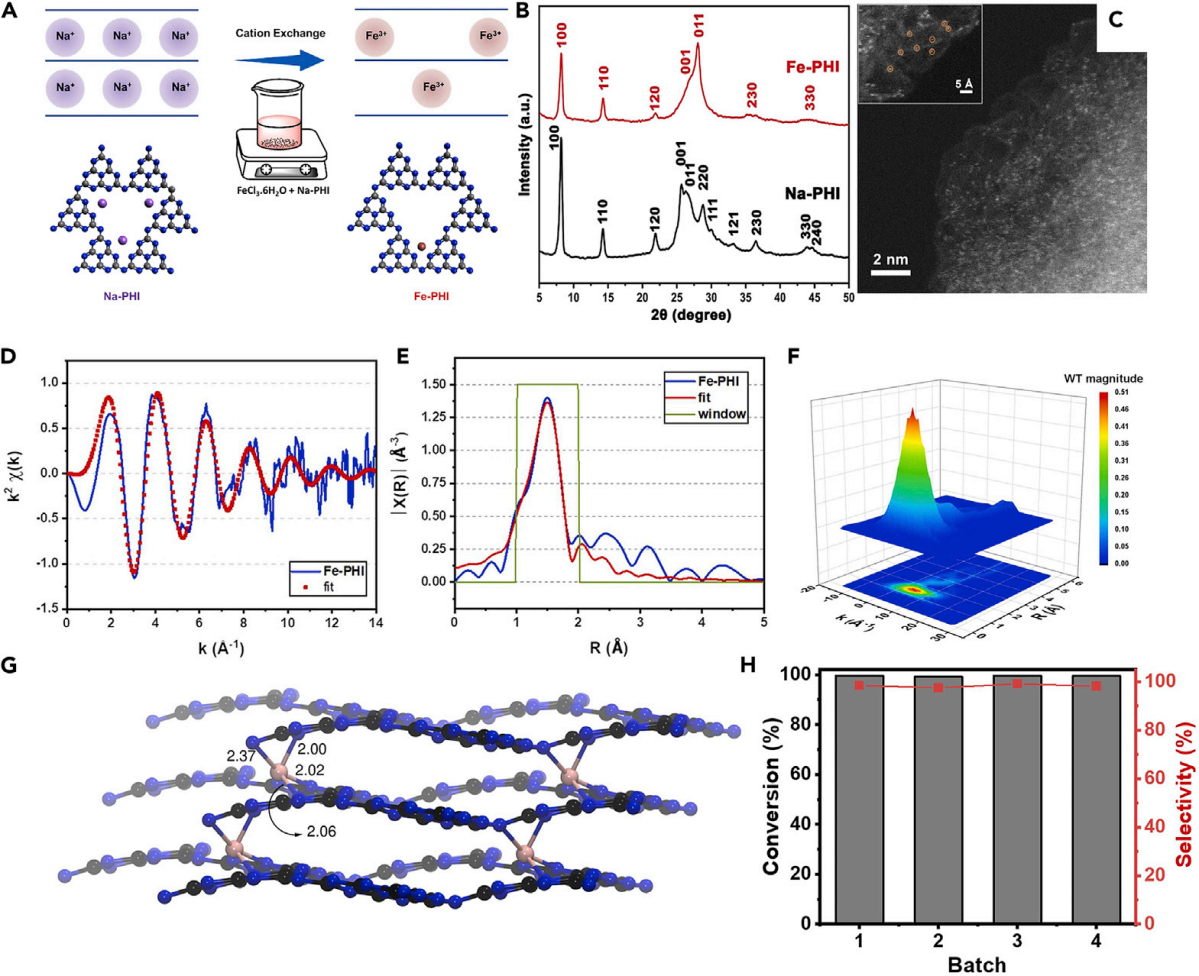
Single-atom photocatalysts for biomass reforming reactions (A–H) (A) The synthesis method of Fe-PHI, (B) XRD patterns for Na-PHI and Fe-PHI, (C) HAADF-STEM image of Fe-PHI, (D) Fe K-edge EXAFS, shown in k_2_-weighted k-space, (E) Fe K-edge EXAFS, shown in k_2_-weighted *R*-space, (F) WT for k_2_-weighted EXAFS signal, (G) DFT simulation of the atomic structure of Fe-PHI (C: black, N: blue, and Fe: pink). (H) Conversion and selectivity of ethylbenzene for different batches of Fe-PHI (0.1%) SAP. Reproduced with permission from Ref (da Silva et al., 2022). Copyright 2022, Elsevier.

Besides the inorganic photocatalytic reaction, SAPs can also be applied in some organic-related photocatalytic reactions, such as biomass reforming, organic synthesis, and pollutant degradation, which were summarized and listed in Table 2. For instance, da Silva et al. mixed Na-PHI and FeCl_3_ together to introduce Fe^3+^ into the poly(heptazine imides) (PHI) matrix, thereby obtaining the target catalyst Fe-PHI (Figure 10A) (da Silva et al., 2022). In Figure 10B, the Fe-PHI XRD peaks were mainly significant differences between 25° and 30°. This apparent difference was assigned to the statistical positioning of Fe ions in the mainframe of the crystal. In Figure 10C, Fe SAs could be well distinguished, confirming the successful synthesis of the Fe-PHI catalyst. EXAFS, Fourier transforms (FTs), and wavelet transforms (WTs) spectra were shown in Figures 10D–10F. Detailed analysis showed that once Fe^3+^ was introduced into the PHI structure, Fe^3+^ would combine with the N atom of the heptazine ring. The DFT calculations showed that Fe^3+^ ions were located between the PHI layers, with each Fe^3+^ ion coordinating with four N atoms and two in each PHI layer (Figure 10G). Moreover, earlier, it was claimed that the C-H bond had high dissociation energy and the C-H bonds were easier to be over-oxidized and lead to the side reactions (Liu et al., 2017). However, it was found that the geometric structure enabled the Fe-PHI catalyst to promote the selective oxidation of C-H bonds. As a result, the Fe-PHI SAP was applied for the catalytic oxidation of the ethylbenzene. It was demonstrated that the Fe-PHI (0.1%) exhibited the superior oxidation activity (Figure 10H), with the 99.6% ethylbenzene conversion rate and 98.4% acetophenone selectivity.

**Table 2:** Comparison list of recently reported SAPs for organic-related photocatalytic reactions

Reaction	SAP	SA	Light intensity	Conversion	Ref.
Benzene oxidation	Cu/C_3_N_4_	Cu	300 W Xe lamp (λ > 420 nm)	92.3% (Phenol)	(Xiao et al., 2020)
Alkenes sulfonation	CNH	Fe	blue LED (460 nm)	94% (β-ketosulfones)	(Wen et al., 2020)
Formic acid dehydrogenation	Co-P_3_/CdS	Co	300 W Xe lamp (λ > 420 nm)	102.9 mmol g^−1^ h^−1^	(Zhou et al., 2020b)
4-Iodoanisole dehalogenation	Ag/AgF	Ag	425 nm LED	92% (Biphenyl derivative)	(Wu et al., 2019b)
Toluene degradation	Au-W/T	Au	350 nm LED	95.4%	(Wang et al., 2022)
HMF oxidation	Cu SAs/p-CNS	Cu	300 W Xe lamp (λ > 400 nm)	77.1%	(Wang et al., 2021a)

**Figure 11:**
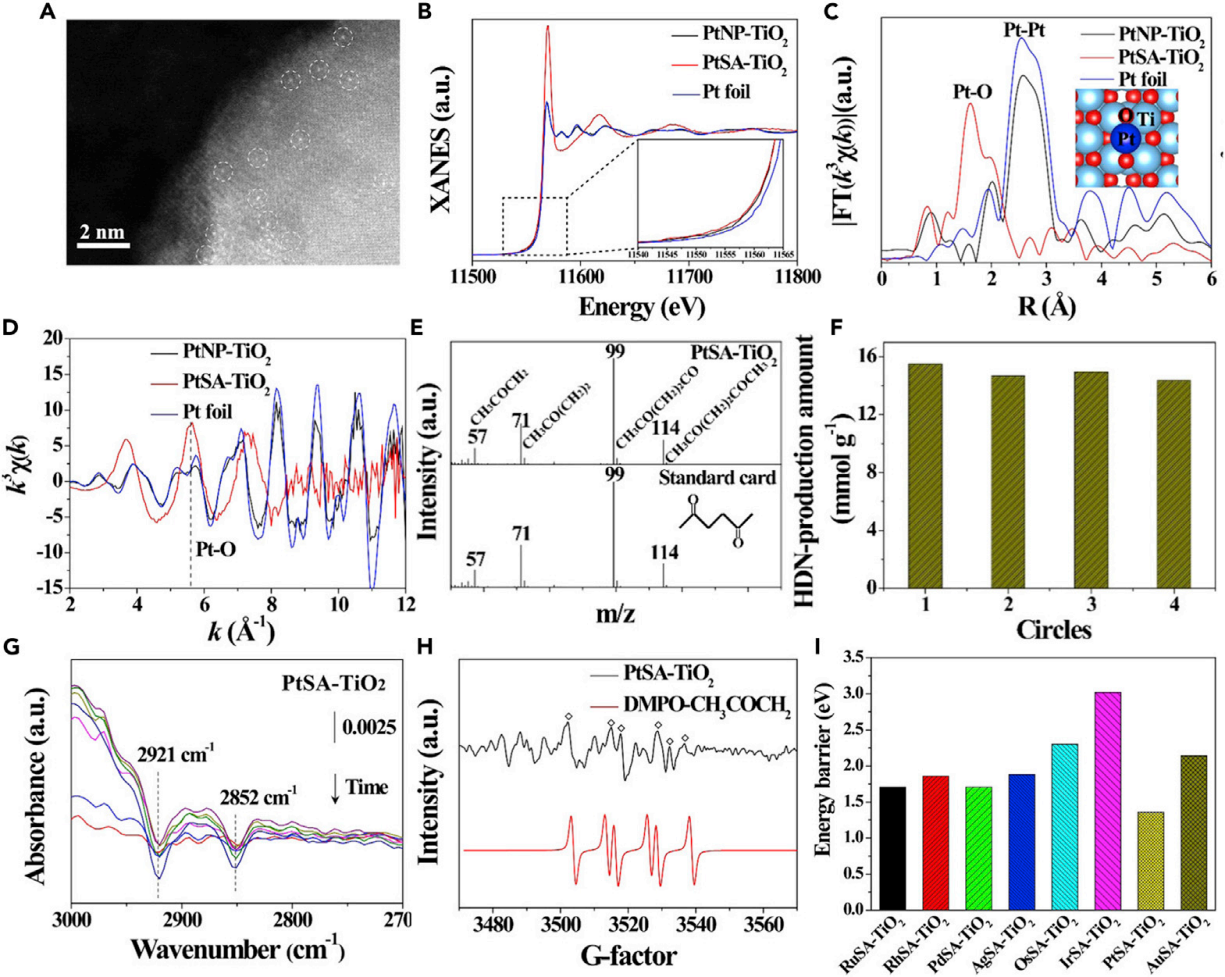
Single-atom photocatalysts for C-C coupling reaction (A–I) (A) HAADF-STEM image of PtSA-TiO_2_. (B) Pt L_3_-edge XANES spectra, the corresponding *k*^3^-weighted (FT) spectra at (C) *R*-space and (D) *k*-space. (E) The GC-MS signals of products over PtSA-TiO_2_. (F) Recycle stability of PtSA-TiO_2_ SAP in 16 h. (G) *In situ* IR spectrum analysis of photocatalytic HDN production over PtSA-TiO_2_. (H) *In situ* ESR spectrum analysis of photocatalytic HDN production. (I) Energy barrier on MSA-TiO_2_ (M = Ru, Rh, Pd, Ag, Os, Ir, Pt, and Au). Reproduced with permission from Ref (Zhou et al., 2020a). Copyright 2020, American Chemical Society.

In addition to the aforementioned biomass refining reaction, the researchers put another focus on organic synthesis to promote the C-C coupling reaction (Toe et al., 2021). Zhou et al. successfully synthesized Pt SAs-loaded TiO_2_ (PtSA-TiO_2_) and applied it for the production of 2,5-hexanedione (HDN), an important chemical in biofuels and medicinal chemistry, from low-cost acetone dehydrogenation (Zhou et al., 2020a). It was the first application of the *in-situ* icing-assisted photocatalytic reduction method to anchor Pt SAs on TiO_2_. The HAADF-STEM image shown in Figure 11A confirmed the presence of Pt SAs on TiO_2_. The coordination structure of Pt SAs on TiO_2_ was analyzed by X-ray absorption near edge structure (XANES) spectra in Figure 11B, which indicated that the absorption edge of Pd SAs was higher than that of Pt nanoparticles-loaded TiO_2_ (PtNP-TiO_2_) and Pt foil (Figure 11B). The EXAFS spectrum of the PtSA-TiO_2_ showed a main peak at 1.61 Å in the R space and a maximum at 5.61 Å^−1^ in the k space (Figure 11C), both of which were assigned to the Pt-O bond (Figures 11D and 11E). Subsequently, the photocatalytic acetone conversion was carried out under the irradiation of a 300 W xenon lamp at 25°C. The results showed that the HDN production rate of PtSA-TiO_2_ was 3.87 mmol g^−1^ h^−1^, which was 6 times higher than that of PtNP-TiO_2_, confirming the excellent reaction activity achieved by the Pt SA catalysts. The gas chromatography-mass spectrometry (GC-MS) in Figure 11E confirmed that the photocatalytic product contained HDN and H_2_. In addition, the HDN-production activity of PtSA-TiO_2_ can maintain four cycles in 16 h (Figure 11F). To further explore the catalysis mechanism, the attenuated total reflection infrared ((ATR)-IR) spectrum discovered that the two IR peaks at 2921 and 2852 cm^−1^ were ascribed to the C-H bond in the methyl group of acetones. As for the PtSA-TiO_2_, these two peaks revealed a sharp decrease (Figure 11G), which implied the activation of methyl and acetone tended to be dehydrogenated at the surface of PtSA-TiO_2_. In Figure 11H, the electron spin resonance (ESR) spectrum exhibited the CH_3_COCH_2_^•^ radical on PtSA-TiO_2_, which was an important intermediate for the production of HDN by C-C coupling. This result showed that Pt SAs exhibited a significant influence on the acetone dehydrogenation reactions. All of the aforementioned results suggested that the PtSA-TiO_2_ possessed a relatively low reaction barrier for acetone dehydrogenation reaction, which was also proved in Figure 11I. Similarly, Wang et al. successfully synthesized Pt/gC_3_N_4_ SAPs using a photo-deposition method, combining the oxidation of benzaldehydes with simultaneous proton reduction (Wang et al., 2021c). The benzaldehyde conversion rate of Pt/gC_3_N_4_ reached 49.5 mmol/g_Pt_, and the hydrogen evolution rate of Pt/gC_3_N_4_ was 24 mmol/g_Pt_. Pt/gC_3_N_4_ SAPs exhibited nearly 100% efficiency per atom in the production of benzoic acid and clean H_2_ fuel.

### The substrate and SAs species

SAPs usually consist of two parts, the supporting substrate and the SAs. By decorating the substrate materials with SAs, enhanced photocatalytic performance could always be obtained. Therefore, in the following section, the current research progress of both the substrate and SAs will be comprehensively overviewed.

#### Different substrates of SAPs

To synthesize SAPs, the applied supporting substrates are usually semiconductor materials. Meanwhile, acting as the substrates of SAPs, the applied semiconductors should be able to anchor the SAs and prevent the aggregation of the SAs. In this section, the supporting substrate catalysts will be categorized as organic, inorganic, and carbon-based materials.

##### Organic substrates

**Figure 12:**
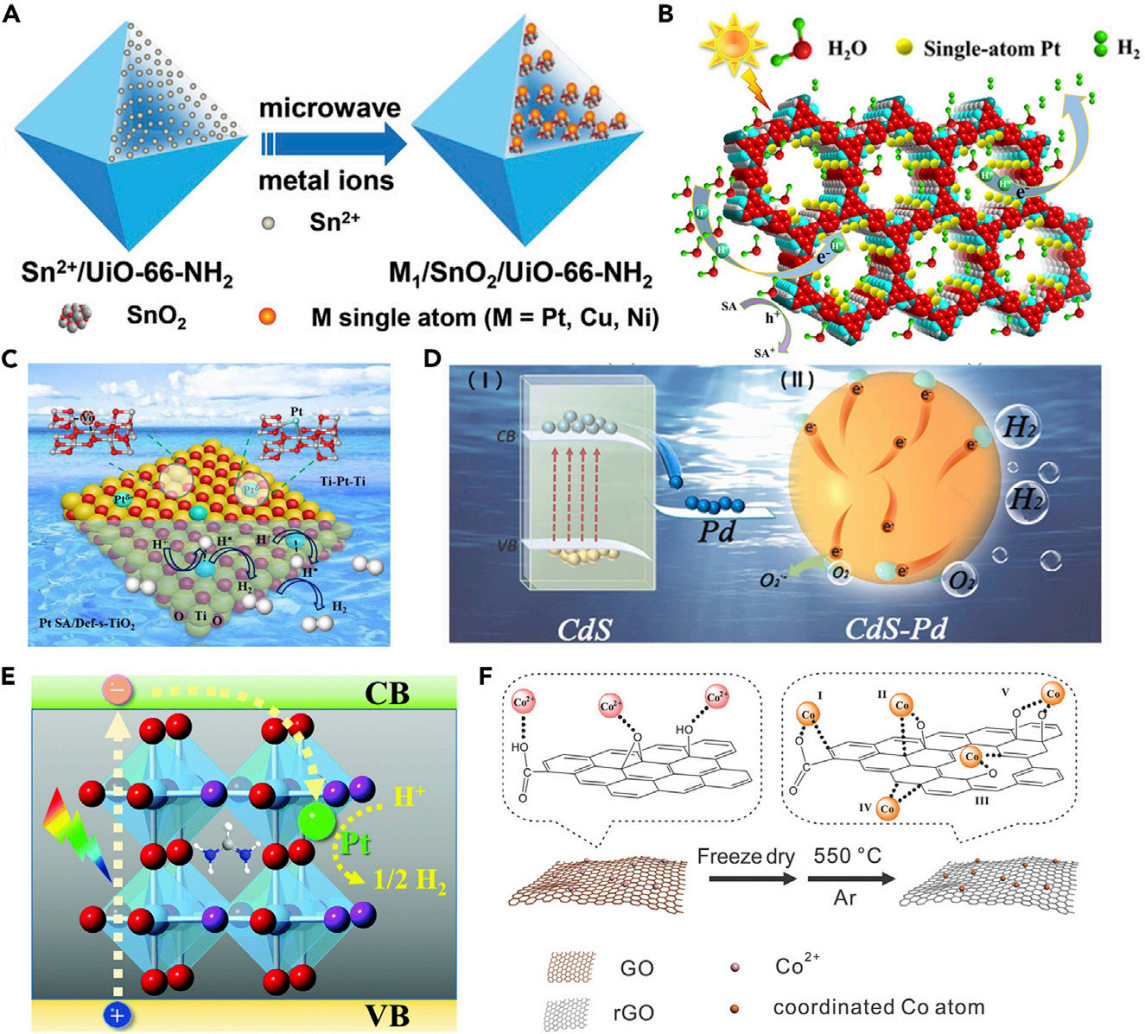
Different substrates for the synthesis of single-atom photocatalysts (A) Schematic illustration of metal/SnO_2_/UiO-66-NH_2_. Reproduced with permission from Ref (Sui et al., 2021). Copyright 2021, Wiley. (B) Pt single-atom anchored on TpPa-1-COF over water splitting. Reproduced with permission from Ref (Dong et al., 2021a). Copyright 2021, American Chemical Society. (C) Pt SA/Def-s-TiO_2_ for photocatalytic H_2_ evolution. Reproduced with permission from Ref (Hu et al., 2021b). Copyright 2021, Elsevier. (D) Pd single-atom on CdS over water splitting. Reproduced with permission from Ref (Li et al., 2022). Copyright 2022, Elsevier. (E) Pt/FAPbBr_3-x_I_x_ for H_2_ evolution. Reproduced with permission from Ref (Wu et al., 2022). Copyright 2022, Royal Society of Chemistry. (F) Co_1_-G catalyst synthetic procedure. Reproduced with permission from Ref (Gao et al., 2018). Copyright 2018, Wiley.

Currently, organic materials have been widely used as SAPs substrates, such as metal-organic frameworks (MOFs) and covalent organic frameworks (COFs). For the MOF substrates, the selection criteria are mainly based on the following three aspects (Jiao and Jiang, 2019; Li et al., 2016): (1) possessing large specific surface area, which is conducive to the adsorption of reactants; (2) exhibiting optical activity, which can generate photo-induced carriers to participate in the photocatalytic reaction; and (3) providing pore confinement, which can prevent the aggregation of metal with a relatively high metal loading. For MOF itself, its unsaturated coordination sites, defects, and the porous structure can be utilized to anchor metal SAs, making it an ideal substrate for anchoring the SAs (Jiao and Jiang, 2019). For example, the Pt_1_/SnO_2_/UiO-66-NH_2_ catalysts were successfully synthesized by Sui et al., applying for the visible-light-driven HER (Sui et al., 2021). The obtained Pt_1_/SnO_2_/UiO-66-NH_2_ SAPs showed a superior H_2_ evolution rate of 2167 μmol g^−1^ h^−1^. Further, Li et al. synthesized MOF-808-EDTA with implanted Pt SAs (Li et al., 2019), which exhibited an excellent photocatalytic H_2_ evolution rate (68.33 mmol g^−1^ h^−1^) under visible light irradiation (Figure 12A).

As for the COFs, SAs can be confined within the COF through the coordination interaction between the metal atom and the binding groups in COF (Wei et al., 2020). Moreover, COFs possess heteroatom-rich pore walls that can facilitate reactant adsorption and charge transfer, resulting in more efficient photocatalytic reactions (Zeng and Xue, 2021). Therefore, the utilization of COFs as the substrates to capture SAs is expected to bring new opportunities for the development of SAPs. For example, Dong et al. reported a two-dimensional β-ketoenamine-linked COF supporting Pt SAs (Pt_1_@TpPa-1) for photocatalytic HER (Dong et al., 2021a). TpPa-1-COF showed special holes and dispersed unsaturated coordinating nitrogen atoms, which made the Pt SAs highly dispersed. The optimal 3% Pt_1_@TpPa-1 showed the best H_2_ evolution rate of 99.86 mmol g_Pt_^−1^ h^−1^ (Figure 12B). Besides Pt, the active Mo SAs were also impregnated in the TPBPY-type COF to get Mo-COF, realizing an efficient photocatalytic reduction of CO_2_ to produce C_2_H_4_ (3.57 μmol g^−1^ h^−1^) (Kou et al., 2021).

##### Inorganic substrates

To date, metal oxides are the most used inorganic substrate for the synthesis of SAPs, as the SAs can be anchored on metal oxides through the metal-oxygen bonds or be stabilized through oxygen vacancies, contributing to the enhanced stability of the SAPs. For instance, Hu et al. demonstrated that the Pt SAs could incorporate defective TiO_2_ nanosheets (Pt SA/Def-s-TiO_2_) for photocatalytic water splitting (Figure 12C) (Hu et al., 2021b). The surface oxygen vacancies could efficiently stabilize the Pt SAs by forming a three-center Ti-Pt-Ti structure, which also contributed to the enhanced charge transfer processes. As a result, greatly enhanced photocatalytic HER was evidenced. Notably, the Pt SA/Def-s-TiO_2_ SAPs exhibited an enhanced H_2_ evolution performance (13460.7 μmol h^−1^ g^−1^), which was 29.0 times higher than that of TiO_2_ nanosheets.

Similar to metal oxides, the unsaturated coordinated sulfur atoms in metal sulfides could also bond with metal SAs to form SAPs. For instance, Li et al. synthesized CdS-Pd SAPs through the photoreduction method (Li et al., 2022). It was demonstrated that the CdS-Pd SAPs exhibited considerable structural stability and photocatalytic HER performance due to the synergistic interaction between CdS and Pd, achieving an efficient charge transfer process to the catalysts’ surface. The obtained H_2_ evolution rate (947.93 μmol g^−1^ h^−1^) was about 110 times higher than that of pure CdS NPs (8.64 μmol g^−1^ h^−1^).

Besides the metal oxides and sulfides, recently, halide perovskites materials are also demonstrated to be a potential substrate material to synthesize the SAPs. Halide perovskites possess fascinating properties such as broad light absorption, long charge carrier migration lengths, etc. (Fu and Draxl, 2019). Currently, the halide perovskites are demonstrated with excellent photocatalytic performance, besides being applied in the photovoltaic fields. In this regard, synthesizing halide perovskites-based SAPs is promising to obtain extraordinary catalytic performance. The presence of SAs is expected to effectively enhance the interaction between the halide perovskite and the reactant molecules (Fu and Draxl, 2019). For instance, Wu et al. successfully anchored Pt SAs onto FAPbBr_3-x_I_x_ (Pt/FAPbBr_3-x_I_x_) with high dispersibility and stability (Figure 12E) (Wu et al., 2022). The obtained Pt/FAPbBr_3-x_I_x_ SAPs showed enhanced photocatalytic hydrogen production activity, reaching 682.6 μmol h^−1^ (100 mg). In addition, Hu et al. demonstrated that the Pt SAs could be deposited onto the CsPbBr_3_ NCs (Pt-SA/CsPbBr_3_) through the formation of Pt-O and Pt-Br bonds (Hu et al., 2021a). Compared with pristine CsPbBr_3_ NCs, the trap levels exhibited in the Pt-SA/CsPbBr_3_ were ascribed to the deposition of Pt SAs, leading to an enhanced separation capability of the photogenerated carriers. Because of the fast carrier transfer from CsPbBr_3_ to Pt SAs, the Pt-SA/CsPbBr_3_ exhibited a superior activity toward the photocatalytic propyne semi-hydrogenation (TOF = 122.0 h^−1^).

##### Carbon-based substrates

Because of the excellent conductivity of graphene, carbon-based substrates have been widely used to anchor metal SAs for not only electrocatalysis (Su et al., 2021a; Su et al., 2021b; Tian et al., 2021; Wang et al., 2021f) but also the photocatalysis field (Zhuo et al., 2020). Similar to organic and inorganic substrates, structurally modified graphene can bind with SAs through the coordination interactions with oxygen- or nitrogen-containing functional groups. For instance, Gao et al. used oxidized graphene nanosheets as the substrates to immobilize the isolated Co SAs (Co_1_-G). Under this circumstance, the graphene acted as a bridge to connect the Ru(bpy)_3_ photosensitizer and the Co SAs, thereby realizing effective charge transfer and CO_2_ reduction (Gao et al., 2018). It was demonstrated that the Co SAs were coordinated with the carbon and residue oxygen on the graphene surface and exhibited outstanding TON (678) and TOF (3.77 min^−1^) toward photocatalytic CRR. In addition, N-doped carbon substrates are also widely applied in photocatalysis, which provide rich coordination sites for the anchoring of the SAs (Liu et al., 2021b). For instance, Zhao et al. demonstrated that the Ni SAs-decorated N-graphene/CdS (Ni-NG/CdS) could be efficient SAPs for photocatalytic HER (Zhao et al., 2018). In this work, Ni SAs were anchored on the vacancies in nitrogen-doped graphene (Ni-NG). In the obtained catalysts, the Ni-NG acted as the electron storage medium to suppress the carrier recombination and the active site for the reduction reaction. As a result, the Ni-NG/CdS received an outstanding photocatalytic HER performance with a quantum efficiency of 48.2% at 420 nm.

#### Different SAs of SAPs

As discussed earlier, to regulate the charge carriers’ generation/transfer and surface reaction process, loading metal nanoparticles to modify the pristine semiconductor catalysts is a generally applied strategy. However, due to the high expense and scarce reserves of noble metal, increasing the utilization efficiency of metal atoms is of great importance, which is also applicable to nonnoble metal species (Li et al., 2021b). In the following section, the currently studied SAs species are systematically summarized.

##### Noble metal SAs

**Figure 13:**
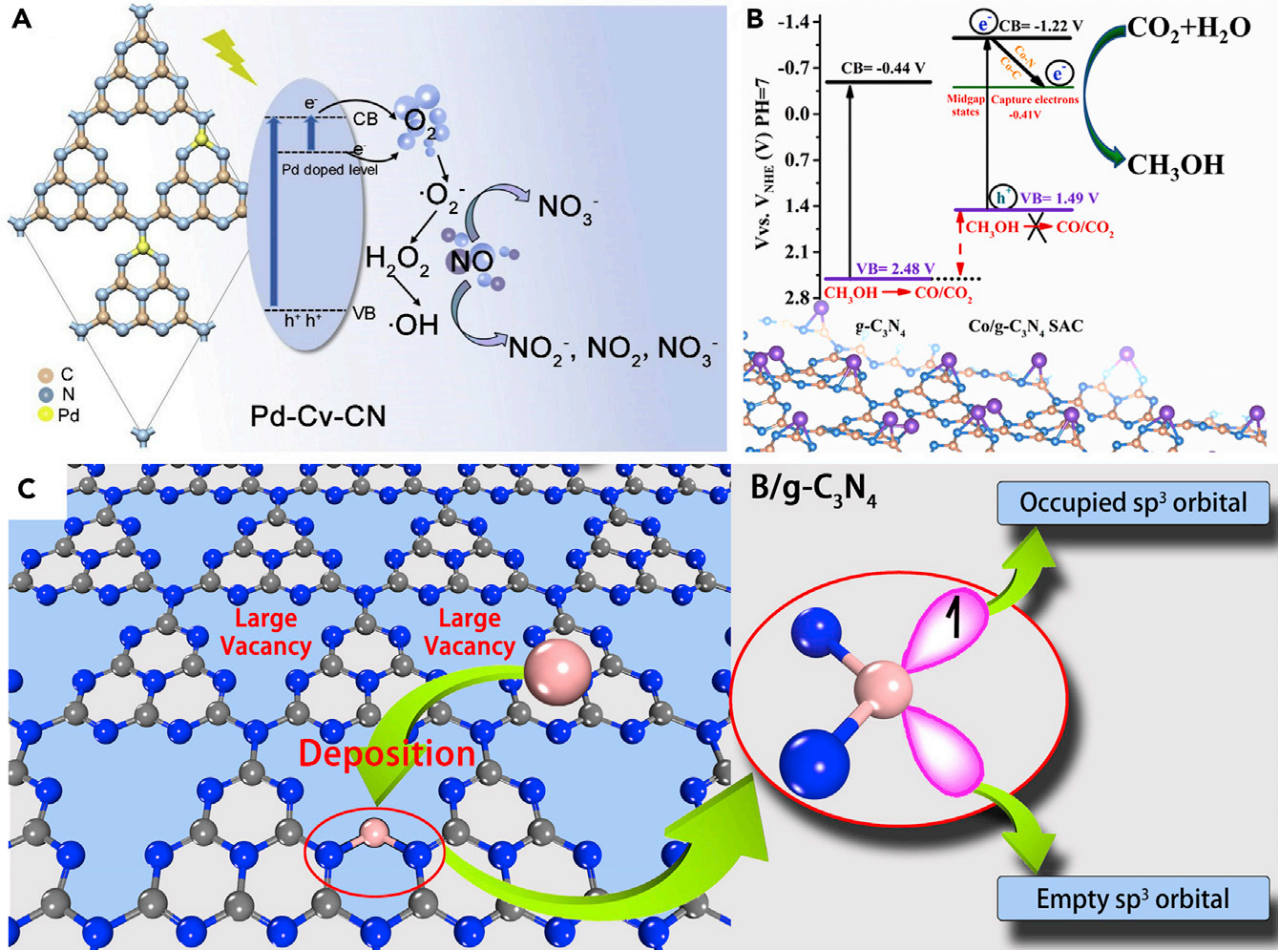
Different single-atom species for single-atom photocatalysts (A) Schematic illustration of the surface reaction mechanism of photocatalytic oxidation of NO over Pd-Cv-CN. Reproduced with permission from Ref (Liu et al., 2021a). Copyright 2021, Elsevier. (B) Boosting photocatalytic CO_2_ reduction to methanol over Co-N_2_C. Reproduced with permission from Ref (Ma et al., 2022). Copyright 2022, Elsevier. (C) N_2_ fixation driven by visible light over B/g-C_3_N_4_. Reproduced with permission from Ref (Ling et al., 2018). Copyright 2018, American Chemical Society.

Currently, various noble metals, such as Pt, Pd, Ir, Au, Ag, Rh, Ru, etc., have been applied to synthesize SAPs due to their excellent catalytic activities. For example, Liu et al. applied g-C_3_N_4_ with carbon vacancies (Cv-CN) to anchor Pd SAs (Pd-Cv-CN), applying for the photocatalytic NO reduction reaction (Figure 13A) (Liu et al., 2021a). The results showed that the Pd SAs could be successfully anchored to the carbon vacancies and uniformly dispersed on the Cv-CN surface, thereby forming isolated Pd-N_3_ sites. In the case of photocatalytic NO conversion, Pd-Cv-CN not only exhibited higher conversion efficiency of 56.3% but also higher selectivity and stability toward NO_3_^−^ generation compared with Cv-CN. Similarly, Li et al. prepared Pd/TiO_2_ SAPs by the liquid-phase reduction method and applied it in the photocatalytic CRR (Li et al., 2017). The results showed that the Pd SAs could be uniformly dispersed on the surface of TiO_2_, leading to improved CRR activity. The enhanced CRR efficiency was attributed to the synergistic effect of Pd SAs and TiO_2_, as the Pd SAs could act as the electron trap center to capture photogenerated electrons and inhibit the recombination of photo-induced electrons and holes.

##### Nonnoble metal SAs

The nonnoble metal-based SAPs are focused on the transition metals such as Fe, Co, Cu, Ni, etc. The transition metals have vacant orbitals that can accept electrons as electron traps and avoid the recombination of photogenerated electron-hole pairs (Abdullah et al., 2017). Ma et al. dispersed Co SAs on g-C_3_N_4_ nanosheets with ultra-high density of Co-N_2_C active sites and applied the obtained SAPs for the photocatalytic CRR (Figure 13B) (Ma et al., 2022). They demonstrated that the Co-N_2_C sites served not only as the electron aggregation center but also as the CO_2_ adsorption/activation sites, which subsequently promoted the photocatalytic methanol generation performance. As a result, the methanol formation rate for 4 h was 941.9 μmol g^−1^ over Co/g-C_3_N_4_-0.2, which was 13.4 times of g-C_3_N_4_ (17.7 μmol g^−1^). Moreover, Zhang et al. dispersed Co SAs into MOFs (MOF-525-Co) for the CO_2_ photoreduction (Zhang et al., 2016). They proved that the Co SAs could act as the CO_2_ adsorption sites. Simultaneously, the photogenerated electrons could transfer from the MOFs to the Co active sites feasibly, thereby improving the photocatalytic efficiency.

##### Metal-free SAs

With empty and occupied orbitals, the atomic structures of some nonmetal elements (e.g., B, Si, etc.) are similar to that of the transition metals (Zhao et al., 2022). Compared with metal-based SAPs, metal-free-based SAPs have also been extensively studied due to their low cost and environmental friendliness. Although metal-free-based SAPs show weaker catalytic activity compared with metal-based SAPs, they yet have good stability and strong resistance to poisoning and deactivation. Ling et al. designed a boron-atom-decorated graphitic-carbon nitride (B/g-C_3_N_4_) for the photocatalytic NRR (Figure 13C) (Ling et al., 2018). By analyzing the extranuclear electronic structure of boron atoms, they found that the sp^3^-hybridized boron atoms were similar to transition metals with empty and occupied orbitals, which could be used as the reaction active center for the NRR. Furthermore, the modification of B SAs can significantly enhance the visible light absorption capability of g-C_3_N_4_, thus promising to realize the visible-light-driven NRR. Lv et al. also reported that B SAs could be applied to reduce dinitrogen to ammonia spontaneously (Lv et al., 2019).

### Characterization of SAPs

Generally, for SAPs, the metal/nonmetal species are dispersed on the supporting substrates in the form of SAs, acting as the active sites for the photocatalytic reaction. Therefore, it is crucial to clarify the geometric structure, electronic structures, and the spatial distribution of SAs for the deep study of SAPs. Advanced electron microscopy analysis techniques, such as scanning tunneling microscope (STM) and high-angle annular dark-field-scanning transmission electron microscope (HAADF-STEM), can provide advanced understandings of the structure of SAPs, making it possible to identify the SAs at the magnitude at *c.a.* ∼ 0.1 nm; spectroscopy techniques, such as X-ray absorption fine structure (XAFS) spectroscopy and infrared (IR) spectroscopy, can also be applied to identify the existence of SAs and clarify the electronic structure and chemical state of the obtained SAPs.

#### Microscopic techniques

**Figure 14:**
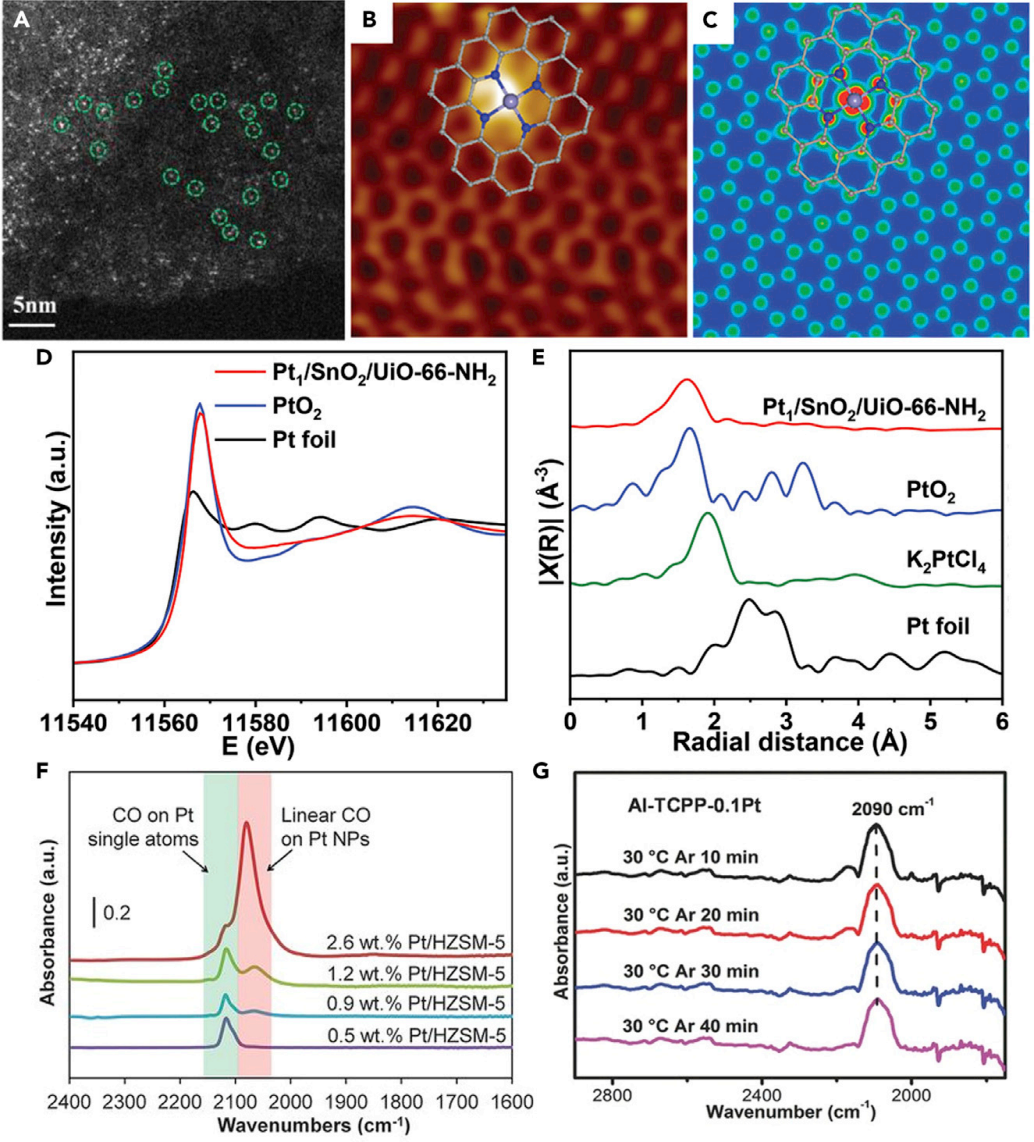
Characterization of single-atom photocatalysts (A) The spherical-aberration-corrected HAADF-STEM images. Reproduced with permission from Ref (Chen et al., 2020a). Copyright 2020, American Chemical Society. (B–C) Low-temperature STM image of FeN_4_/GN (The gray, blue, and light blue balls represent C, N, and Fe atoms, respectively), and (C) simulated STM image for (B). The inserted schematic structures represent the structure of the graphene-embedded FeN_4_. Reproduced with permission from Ref (Deng et al., 2015). Copyright 2015, Science. (D–E) The Pt L_3_-edge XANES spectra for Pt_1_/SnO_2_/UiO-66-NH_2_, PtO_2_, and Pt foil, and (e) Fourier transformed (FT) k^2^-weighted χ(k)-function of the EXAFS spectra for Pt_1_/SnO_2_/UiO-66-NH_2_, PtO_2_, K_2_PtCl_4_, and Pt foil. Reproduced with permission from Ref (Sui et al., 2021). Copyright 2021, Wiley. (F) IR spectra of CO adsorbed on different Pt/HZSM-5 after the desorption processes. Reproduced with permission from Ref (Ding et al., 2015). Copyright 2015, Science. (G) DRIFTS spectra of CO adsorbed on Al-TCPP-0.1Pt after being purged with Ar gas for different times. Reproduced with permission from Ref (Fang et al., 2018). Copyright 2018, Wiley.

The typical electron microscopes techniques, scanning electron microscope (SEM) and transmission electron microscope (TEM), can toughly identify the SAs at the atomic level. In this regard, HAADF-STEM is applied to observe SAs due to the improved spatial resolution of its sub-Angstrom probe (Gao et al., 2019b; Peng et al., 2004). This technique has been chosen for heavy elements on light substrates for the strong correlation between atomic number and imaged intensity, called Z-contrast (LeBeau et al., 2008; Nellist et al., 2010). Under dark-field conditions, different atoms have different Z-contrasts, making the atoms distinguishable by observing their brightness in the HAADF-STEM images (Chung et al., 2019). In Figure 14A, the spherical-aberration-corrected HAADF-STEM images of O/La-CN SAPs showed the bright dots, which corresponded to the even dispersed La SAs on CN substrate due to the different Z contrasts of La, C, and N atoms (Chen et al., 2020a).

STM is a characterization technique applied to probe the surfaces and absorb substances at the atomic level with ultra-high resolution of 0.1 nm laterally and 0.01 nm in depth. The SAs can be imaged and manipulated with the conductive tips (Gao et al., 2020a). For example, Deng et al. used STM to reveal the existence of Fe SAs with FeN_4_ center in the graphene matrix as shown in Figure 14B (Deng et al., 2015). The iron center was shown as a bright spot, whereas adjacent atoms (C and N) exhibited a higher apparent height than other C atoms in the graphene matrix. In the simulated STM images, the FeN_4_ centers embedded in the graphene lattice were consistent with the STM images, which better revealed the iron centers significantly alter the density of states of adjacent atoms (N and C) (Figure 14C).

#### Spectroscopic techniques

In addition to the aforementioned microscopy techniques, XAFS spectroscopy, including XANES and EXAFS, is another effective way for the characterization of SAPs, which is used to analyze the coordination environment and electronic structure in the material structure. According to the characteristics of peaks and shoulders in the XANES spectrum, the electronic structure and chemical valence state of SAs can be obtained. In the EXAFS spectra, SAs can be identified through morphological imaging characterization and corresponding spectral information sensitive to atomic structure, so as to obtain the coordination number, coordination form, and coordination distance of the planted SAs to the adjacent atoms in the SAPs. For instance, Sui et al. performed XAS to determine the coordination environment and chemical state of Pt species in Pt_1_/SnO_2_/UiO-66-NH_2_ SAPs (Sui et al., 2022). From the L_3_-edge image, it could be seen that the peak intensity of Pt_1_/SnO_2_/UiO-66-NH_2_ was closer to that of PtO_2_, implying the presence of a highly oxidized Pt state (Figure 14D). The Fourier transform expansion X-ray absorption fine structure spectra (FT-EXAFS) of the Pt_1_/SnO_2_/UiO-66-NH_2_ gave only a dominant peak at about 1.63 Å, which can be attributed to the first shell of the Pt-O bond, rather than the Pt-Cl bond and Pt-Pt bond, suggesting the existence of atomically dispersed Pt sites in Pt_1_/SnO_2_/UiO-66-NH_2_ (Figure 14E).

IR spectroscopy can also be utilized to identify the presence of SAs and to quantify the percentage of metal SAs to some extent (Chen et al., 2017b; Liu, 2017). The principle is as follows, IR is used to detect the interaction between the substrate and the adsorbed molecule by utilizing probe molecules (e.g., CO, NH_3_, pyridine, etc.) (Gallenkamp et al., 2021). For instance, Ding et al. applied the IR spectra to confirm the Pt SAs in the Pt/HZSM-5 catalysts by analyzing the CO adsorption mode (Ding et al., 2015). As shown in Figure 14F, the peak at 2115 cm^−1^ was attributed to CO molecules adsorbed on Pt SAs. Meanwhile, the peak at 2090 cm^−1^ was ascribed to CO molecules linearly adsorbed on Pt nanoparticles. It could be inferred that Pt existed as SAs on Pt/HZSM-5 with a low Pt loading at 0.5 wt % by studying the changes in peak intensity for the four catalysts. And the Pt atoms tended to agglomerate to form Pt nanoparticles after increasing the Pt loading from 0.5 wt% to 2.6 wt%. In addition, diffuse reflectance infrared Fourier transform spectroscopy (DRIFTS) offers another technique to gain insight into the local information of the SAPs, as it is usually applied to *in-situ* collect information of the surface reactive species and intermediates during the reaction (Yang et al., 2019). Typically, CO is used as the probe molecule in DRIFTS studies because of its advantages in characterizing the exposed noble metal sites on loaded catalysts (Liang et al., 2022). For instance, Fang et al. collected the *in-situ* DRIFTS spectra of the CO adsorption behavior over the Al-TCPP-0.1Pt SAPs (Fang et al., 2018). After purging with Ar to remove gaseous CO, the peak centered at 2090 cm^−1^ corresponded to the CO chemisorbed on Pt SAs (Figure 14G). In the ranges of 2080–2030 cm^−1^ and 1920–1950 cm^−1^, no bands that could linearly and bridge CO adsorption on Pt clusters and nanoparticles appeared, implying that all Pt species were atomically dispersed.

## Conclusion and prospects

To date, SAPs have been widely studied in solar-driven chemicals/fuels generation, with various SAPs synthesis strategies being established. The pioneer works demonstrated that the introduction of SAs to the commonly used semiconductor catalysts can directly influence the overall photocatalysis process: (1) by modulating the band structure with impurity level or directly reducing the bandgap, SAPs, therefore, exhibits greatly enlarged optical absorption range; (2) due to the unique band bending effect at the metal SAs/semiconductor interfaces, the spatial separation and transfer of the photogenerated carriers can be significantly promoted; (3) with the tuneable coordination environment of the SAs, SAPs are equipped with boosted reaction active sites and better product selectivity. Moreover, rationally choosing the supporting substrate materials and the loaded SAs species is expected to regulate the surface reaction process efficiently. However, due to the insufficient understanding of the structure-catalytic performance relationship based on SAPs, in the future, the study of SAPs still faces some crucial issues:

(1) Due to the strong influence of the local electronic structure of the material, the rational design of SAPs with high loading rates of the SAs is still a significant challenge. The knowledge to prepare stable and efficient SAPs with high SAs loading is still in its infancy. The loading amounts of SAs can reach 23 wt% now for nitrogen-doped carbon and polymeric carbon nitride (Lu et al., 2021). However, for other supports, the loading rates are still less satisfactory. Consequently, it will be promising to achieve higher SAs loading rates by ligands protection or through the ambient multistep method to regulate the removal of the ligands from the metal precursors and enhance the associated interactions between SAs and substrates.
(2) To date, there is still lack of efficient methods to monitor the reaction dynamics of the catalytic process. Currently, the characterizations of the ligand environment and associated charge transfer processes mainly rely on theoretical calculations. Direct monitoring of the reaction dynamics is still challenging. In the future study, *in-situ* characterization techniques, such as *in-situ* electron microscopy or *in-situ* synchrotron radiation technique, need to be applied to monitor and probe the photocatalytic reaction process. Combined with theoretical calculations, the relationship between their structure and catalytic performance can be better explained.
(3) The advanced understanding of the photocatalytic mechanism over the SAPs is insufficient, making this technique challenging to practical application. To date, only a few studies tried to uncover the effect of the SAs on the reaction mechanism. To illustrate the impact of SAs on the reaction pathway, it will be promising to apply homogenized substrates for the SAs loading to investigate the accurate active sites of SAPs. This will benefit the design of some specific SAPs fitted for the targeted reactions on an atomic scale.

**Abbreviations**

SACs Single-atom catalysts

SAs Isolated single-atoms

SAPs Single-atom photocatalysts

CVD Chemical vapor deposition

FSP Flame spray pyrolysis

ALD Atomic layer deposition

CB Conduction band

VB Valence band

COFs Covalent organic frameworks

PL Photoluminescence

TRPL Time-resolved PL

TOFs Turnover frequencies

TONs Turnover numbers

FTIR Fourier transform infrared spectroscopy

TPD Temperature-programmed desorption

PHI Poly(heptazine imides)

EXAFS Extended X-ray absorption fine structures

FTs Fourier transforms

WTs Wavelet transforms

HDN 2,5-hexanedione

XANES X-ray absorption near edge structure

GC-MS Gas chromatography-mass spectrometry

ESR Electron spin resonance

HADDF-STEM High-angle annular dark-field scanning transmission electron microscopy

FT-EXAFS Fourier transform EXAFS

HER Hydrogen evolution reaction

CRR Carbon dioxide reduction reaction

NRR Nitrogen reduction reaction

DRIFTS Diffuse reflectance infrared Fourier transform spectroscopy

STM scanning tunneling microscope

XAFs X-ray absorption fine structure

IR Infrared

(ATR)-IR Attenuated total reflection infrared

MOFs Metal-organic frameworks
